# Efficacy and safety of glucocorticoid combined with cyclophosphamide therapy on membranous nephropathy: a systematic review and meta-analysis

**DOI:** 10.3389/fphar.2024.1480638

**Published:** 2024-11-27

**Authors:** Chengcheng Feng, Xuexun Chen, Xiangming Wang, Min Guo, Zhentao Guo

**Affiliations:** ^1^ School of Clinical Medicine, Shandong Second Medical University, Weifang, China; ^2^ Department of Nephrology, School of Clinical Medicine, Affiliated Hospital of Shandong Second Medical University, Shandong Second Medical University, Weifang, China

**Keywords:** glucocorticoid, combination, cyclophosphamide, membranous nephropathy, efficacy, safety, meta-analysis

## Abstract

**Background:**

This review systematically evaluates the efficacy and safety of the combined treatment of glucocorticoids (GC) and cyclophosphamide (CTX) in patients with membranous nephropathy (MN).

**Methods:**

As of June 2024, a comprehensive literature search was performed utilizing several reputable databases, including PubMed, Embase, the Cochrane Library, China National Knowledge Infrastructure (CNKI), and Wanfang. A meta-analysis was then carried out using Review Manager 5.4 and STATA/SE-15 software.

**Results:**

This research evaluated a total of 22 articles involving 1,971 patients. The findings revealed that patients with MN receiving combined GC and CTX therapy had significantly higher complete remission rates (odds ratio = 1.78, *p* = 0.02) and total remission rates (odds ratio = 2.14, *p* = 0.01) when the follow-up period exceeded 12 months. Additionally, this treatment demonstrated greater efficacy in lowering serum creatinine levels compared to the control group (standardized mean difference = −0.19, *p* = 0.04), while its relapse rate was also lower than that of the control group (odds ratio = 0.51, *p* = 0.009). However, it has a high incidence of serious adverse effects (odds ratio = 2.32, *p* = 0.03).

**Conclusion:**

Our systematic review highlights that the combination of GC and CTX demonstrates superior long-term effectiveness and reduced relapse rates in managing membranous nephropathy (MN). Furthermore, this drug combination is considered the optimal choice for normalizing serum creatinine levels. Data on the effectiveness and safety of glucocorticoids alone versus other drugs alone, and the treatment of secondary membranous nephropathy (SMN), are limited.

**Systematic Review Registration:**

https://www.crd.york.ac.uk/prospero/display_record.php?RecordID=566477, identifier CRD42024566477.

## 1 Introduction

In adults, MN represents one of the predominant pathological forms of nephrotic syndrome. MN can be categorized into two types: primary membranous nephropathy (PMN) and secondary membranous nephropathy (SMN), depending on the underlying cause of the condition. PMN, often referred to as idiopathic membranous nephropathy (IMN), typically lacks an identifiable cause. This condition is marked by the thickening of the capillary walls. Immunofluorescence microscopy reveals normal capillary wall cells along with deposits of IgG and C3 situated along the capillary walls. Additionally, subepithelial immune complex deposits can be detected through electron microscopy (Cui et al., 2017). Autoantibodies that target the phospholipase A2 receptor (PLA2R) found in podocytes mediate the disease in 70%–80% of instances. Conversely, in 3%–5% of cases, the disease is driven by autoantibodies targeting thrombospondin type-1 domain-containing 7A (THSD7A) (Beck et al., 2009; Ronco and Debiec, 2020; Tomas et al., 2014). Patients may exhibit symptoms such as massive proteinuria, hypoalbuminemia, edema, and hyperlipidemia (Mingwei et al., 2019). The progression of primary membranous nephropathy (PMN) is variable and challenging to forecast. Research has shown that approximately 20% of patients may achieve spontaneous complete remission, whereas nearly 40% will advance to end-stage renal failure (ESRF). (Mathieson, 1997; Ponticelli, 2007; Schieppati et al., 1993). The currently accepted recommendation advises conservative treatment for IMN patients without manifestations of nephrotic syndrome for 6 months, followed by a decision on immunosuppressive therapy based on remission status. However, the initial treatment for most IMN patients currently involves a combination of glucocorticoids and immunosuppressants, resulting in a remission rate of 70%–80% (Radhakrishnan and Cattran, 2012).

In recent years, there has been a growing use of calcineurin inhibitors, such as cyclosporine A (CsA) and tacrolimus (TAC), in addition to mycophenolate mofetil (MMF) and rituximab (RTX), for the treatment of patients with Idiopathic Membranous Nephropathy (IMN). However, ongoing debate remains about which immunosuppressive regimen is most effective. Alkylating agents and high-dose corticosteroid therapy are associated with significant side effects (du Buf-Vereijken et al., 2004). The research conducted by Cui et al. (2017) indicated that in comparison to the regimen of CTX combined with prednisone, tacrolimus (TAC) combined with prednisone treatment provided more benefits for IMN patients, with the recurrence rate of TAC is not greater than that of CTX, these results align with the findings reported by Chen et al. (2010). However, the study by Fernández-Juárez et al. (2020) indicated that corticosteroid and CTX treatment was more effective in inducing remission compared to TAC and rituximab (RTX) sequential therapy. The former also had a faster rate of remission, with most achieving complete remission, whereas the TAC and RTX groups mostly achieved partial remission. A recent study (Wang et al., 2024) demonstrated that the overall remission rate of CTX combined with GC treatment for IMN (93.48% vs. 78.26%, *p* < 0.05) was higher than using GC alone. CTX combined with GC treatment can effectively improve the overall remission rate of IMN patients and suppress inflammation and oxidative stress.

There is no conclusive evidence yet as to whether the efficacy of GC combined with CTX in treating MN is higher than using GC alone or other single drugs, or whether GC can be replaced by GC combined with other drugs. Additionally, concerns about the potential renal toxicity of other drugs exist. Recently, many new clinical studies addressing this controversy have been published. Consequently, it is essential to gather new evidence through meta-analysis and systematic evaluation to further validate the efficacy and safety of GC combined with CTX in the treatment of patients with MN.

## 2 Materials and methods

### 2.1 Protocol and registration

This meta-analysis is conducted following the latest guidelines for reporting systematic reviews (Page et al., 2021), with the checklist available in Supplementary Table S1. The PROSPERO registration number for this study is CRD42024566477.

### 2.2 Data sources and searches

Computer-assisted searches were performed across databases including CNKI, Wanfang, Web of Science, Embase, PubMed, and the Cochrane Library to identify articles on the combined treatment of GC and CTX for MN, with the search deadline set for June 2024. All articles are in Chinese or English. The Chinese search terms include “cyclophosphamide,” “glucocorticoid,” and “membranous nephropathy.” The English keywords and medical subject terms include: “Glucocorticoid,” “Glucocorticoid Effect,” “Glucocorticoid Effects,” “Sendoxan,” “B 518,” “Cyclophosphamide Anhydrous,” “Membranous Glomerulonephritides,” “Membranous Glomerulonephritis,” and “Membranous Glomerulopathy.” Additionally, we further included one more study by searching for literature related to the control group. The search strategy is detailed in Supplementary Table S2.

### 2.3 Inclusion and exclusion criteria

Inclusion Criteria:

1. Study Design: Case-control studies, clinical trials, cohort studies, and Randomized controlled trials (RCT).
2. Study Population: All patients included in the studies are adults with biopsy-confirmed MN, including both primary and secondary MN, and are aged over 18 years.
3. Intervention/Control: The studies compared the efficacy of GC combined with CTX with GC combined with calcineurin inhibitors (CNIs) such as CsA or TAC, or with CNIs alone, or with GC combined with other drugs such as RTX, leflunomide (LEF), or MMF.
4. Outcomes: Studies reported at least one required outcome on efficacy or safety.
5. The study must evaluate the efficacy of GC combined with CTX for at least 6 months.
6. The study examines the use of medications to support the treatment of patients with MN.

Exclusion Criteria:

1. Animal experiments, review articles, consensus manuscripts, guidelines, meta-analyses, conference records, and case reports are excluded.
2. Studies for which data are not accessible and studies not in English or Chinese.
3. Repetitive publication of data.

### 2.4 Data extraction

Through the retrieval methods mentioned above and the inclusion and exclusion criteria, two independent reviewers collected the data, resolving any discrepancies through consensus.

The data collected included:

1. The time of the study’s publication and the name of the first author.
2. Study design, location, characteristics of the study population, and treatment duration.
3. Number of patients included, age, and gender.
4. Intervention group details: initial dose and administration route of GC, and the dose and administration route of CTX; treatment drugs for the control group.
5. Primary and secondary outcomes: complete remission (CR), total remission (TR), adverse events, proteinuria (g/24 h), serum albumin, serum creatinine, estimated Glomerular Filtration Rate (eGFR), relapse rate (number and duration of relapses), and Anti-phospholipase A2 receptor (Anti-PLA2R) antibody levels. For dichotomous data, extract the number of events and the total number of occurrences. For continuous data, extract the mean and standard deviation before and after the intervention.

### 2.5 Outcome measures

To evaluate treatment efficacy, several indicators should be considered: 1) CR and TR; 2) reduction in proteinuria, as measured in grams per 24 h; 3) improvement in serum albumin levels; 4) changes in eGFR, serum creatinine, and Anti-PLA2R levels; and 5) the relapse rate, which serves as an indirect measure of treatment efficacy.

Additionally, indicators for assessing safety outcomes encompass 1) changes in serum creatinine levels and 2) the presence of adverse events.

CR is defined as proteinuria levels below 0.3 g/24 h, in conjunction with normal serum albumin and normal serum creatinine. PR is defined as a reduction of proteinuria by more than 50% compared to the baseline value; the proteinuria value is less than 3.5 g/24 h, and renal function is stable. TR is calculated by combining CR and partial remission (PR). Detailed definitions of CR and PR in each study are provided in Supplementary Table S3. A relapse occurs when significant proteinuria, surpassing 3.5 g in 24 h, reappears in patients who have previously attained complete remission (CR) or partial remission (PR) and persists for 2 weeks after the removal of triggering factors such as infections. As per the Clavien-Dindo complication grading system, Grade III (necessitating surgery, endoscopy, or radiological intervention), Grade IV (life-threatening complications, such as central nervous system issues requiring intensive care unit care), and Grade V (patient mortality) are categorized as serious adverse events. The studies predominantly highlighted severe adverse events, encompassing severe infections, pneumonia, respiratory infections culminating in sepsis, cancer, avascular necrosis of the femoral head, severe acute kidney injury, and mortality. To evaluate the changes in serum albumin, 24-h proteinuria, serum creatinine, eGFR, and Anti-PLA2R, we will utilize the differences in mean and standard deviation before and after the intervention.

### 2.6 Quality assessment

The quality of each RCT is assessed based on the “risk of bias” evaluated using the Cochrane Collaboration tool, which consists of seven parts. The methodological quality of the study is evaluated in three categories. The quality of non-RCT studies in the meta-analysis is assessed according to the Newcastle-Ottawa Scale (GA Wells et al., 2021). Disagreements between researchers are resolved through consensus.

### 2.7 Statistical analysis

Literature management was conducted using EndNote X9 (Bld 12062), while statistical analysis was performed using Review Manager 5.4 (The Cochrane Collaboration, Nordic Cochrane Center, Copenhagen, Denmark) and STATA/SE-15 (Stata Corp, Texas, United States) software.

For the comparison of binary variables, the odds ratio (OR) is used, while for continuous outcome variables, the standardized mean difference (SMD) is used. The results are reported with a 95% confidence interval (CI) calculated using the Mantel-Haenszel method. Data heterogeneity is evaluated using 95% CI and the *I* ^*2*^ test. If *p* ≥ 0.05 and *I* ^*2*^ < 50%, it suggests no significant heterogeneity. A random effects model is used for data analysis. The threshold for significance in the meta-analysis is established at α = 0.05, with statistical significance indicated by two-sided *p* < 0.05. Sensitivity analysis is conducted using STATA software, and publication bias is assessed using Egger’s test and funnel plots. Furthermore, this study conducts subgroup analyses based on factors such as study design, follow-up, region, population, intervention, and control to explore the stability of results and potential sources of heterogeneity for indicators such as CR, TR, proteinuria, serum albumin, and serum creatinine.

## 3 Results

### 3.1 Literature search and study selection

**FIGURE 1:**
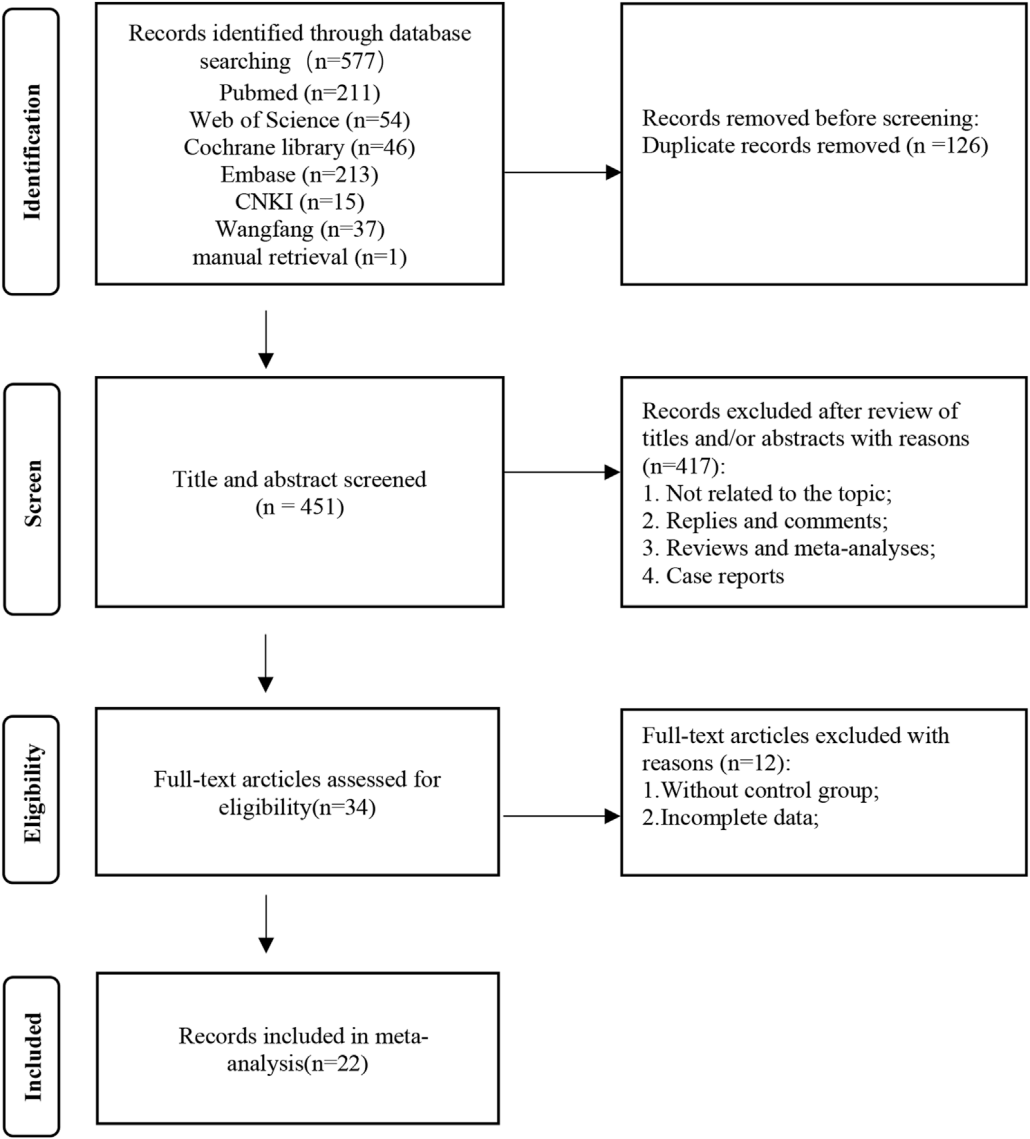
The flow diagram of the systematic retrieval and selection process.

The flow diagram of the systematic retrieval and selection process is shown in Figure 1. A total of 577 relevant articles were identified through systematic literature searches in PubMed (n = 211), Embase (n = 213), Cochrane Library (n = 46), Web of Science (n = 54), CNKI (n = 37), and Wanfang (n = 15), supplemented by a manual search (n = 1). After excluding 126 duplicate articles, the initial screening discarded 451 articles. This leaves 34 articles that require full-text review. Among them, 12 articles were excluded after full reading, resulting in 22 articles that met the inclusion and exclusion criteria (Cui et al., 2017; Mingwei et al., 2019; Fernández-Juárez et al., 2020; Austin et al., 2009; Guo et al., 2020; Hayati et al., 2019; He et al., 2012; Liang et al., 2017; Ramachandran et al., 2016; Ramachandran et al., 2021; Sun et al., 2023; van den Brand et al., 2017; Zou et al., 2019; Qi et al., 2019; Weizhen et al., 2009; Peng et al., 2012; Guo-jian et al., 2015; Zhihu et al., 2021; Weiqing et al., 2016; Lili et al., 2021; Min, 2016; Bianjie and Shengkai, 2021).

### 3.2 Study description

Of the twenty-two included articles, sixteen were prospective randomized controlled trials (RCT) (Mingwei et al., 2019; Fernández-Juárez et al., 2020; Austin et al., 2009; Guo et al., 2020; Hayati et al., 2019; He et al., 2012; Liang et al., 2017; Ramachandran et al., 2016; Ramachandran et al., 2021; Qi et al., 2019; Weizhen et al., 2009; Peng et al., 2012; Zhihu et al., 2021; Lili et al., 2021; Min, 2016; Bianjie and Shengkai, 2021), while six were retrospective clinical studies (Cui et al., 2017; Sun et al., 2023; van den Brand et al., 2017; Zou et al., 2019; Guo-jian et al., 2015; Weiqing et al., 2016). Twenty studies focused on the primary membranous nephropathy population (Cui et al., 2017; Mingwei et al., 2019; Fernández-Juárez et al., 2020; Guo et al., 2020; Hayati et al., 2019; He et al., 2012; Liang et al., 2017; Ramachandran et al., 2016; Ramachandran et al., 2021; Sun et al., 2023; van den Brand et al., 2017; Zou et al., 2019; Qi et al., 2019; Weizhen et al., 2009; Peng et al., 2012; Guo-jian et al., 2015; Weiqing et al., 2016; Lili et al., 2021; Min, 2016; Bianjie and Shengkai, 2021), while two studies focused on the secondary membranous nephropathy population (Austin et al., 2009; Zhihu et al., 2021). Nineteen articles included patients from Asian populations (Cui et al., 2017; Mingwei et al., 2019; Guo et al., 2020; Hayati et al., 2019; He et al., 2012; Liang et al., 2017; Ramachandran et al., 2016; Ramachandran et al., 2021; Sun et al., 2023; Zou et al., 2019; Qi et al., 2019; Weizhen et al., 2009; Peng et al., 2012; Guo-jian et al., 2015; Zhihu et al., 2021; Weiqing et al., 2016; Lili et al., 2021; Min, 2016; Bianjie and Shengkai, 2021), two from European populations (Fernández-Juárez et al., 2020; van den Brand et al., 2017), and one from an American population (Austin et al., 2009). The meta-analysis included a total of 1,971 patients, with 1,077 patients randomized to the GC combined with the CTX group. The follow-up duration in the studies included ranged from 6 to 72 months. Among the 22 articles, one article (Weiqing et al., 2016) was divided into two studies due to different administration methods for GC and CTX, resulting in a total of 23 specific studies. These studies examined the effects of GC in combination with CsA, GC in combination with TAC, GC in combination with MMF, GC in combination with LEF, GC in combination with TAC and RTX, GC in combination with CsA and TAC, single-use TAC, single-use RTX, and single-use CsA in 4 (Austin et al., 2009; Weiqing et al., 2016; Lili et al., 2021; Min, 2016), 9 (1, 19, 21, 22, 25, 27, 29, 30, 34), 2 (18, 28), 1 (17), 1 (12), 1 (23), 2 (20, 26), 1 (24) and 1 (5) articles, respectively. Among the experimental groups, GC combined with CTX was studied in 17 studies (Cui et al., 2017; Mingwei et al., 2019; Austin et al., 2009; Guo et al., 2020; Hayati et al., 2019; He et al., 2012; Liang et al., 2017; Sun et al., 2023; Zou et al., 2019; Qi et al., 2019; Weizhen et al., 2009; Peng et al., 2012; Guo-jian et al., 2015; Weiqing et al., 2016; Lili et al., 2021; Min, 2016; Bianjie and Shengkai, 2021), with GC administered orally in 17 studies and intravenously in 1 study (Zhihu et al., 2021). The administration methods for GC were both oral and intravenous in 5 studies (Fernández-Juárez et al., 2020; Ramachandran et al., 2016; Ramachandran et al., 2021; van den Brand et al., 2017; Weiqing et al., 2016), while for CTX, the administration was oral in 6 studies (Cui et al., 2017; Fernández-Juárez et al., 2020; Ramachandran et al., 2016; Ramachandran et al., 2021; van den Brand et al., 2017; Weiqing et al., 2016) and intravenous in 17 studies (Mingwei et al., 2019; Austin et al., 2009; Guo et al., 2020; Hayati et al., 2019; He et al., 2012; Liang et al., 2017; Sun et al., 2023; Zou et al., 2019; Qi et al., 2019; Weizhen et al., 2009; Peng et al., 2012; Guo-jian et al., 2015; Zhihu et al., 2021; Weiqing et al., 2016; Lili et al., 2021; Min, 2016; Bianjie and Shengkai, 2021). The initial dosage and administration method of GC, the dosage and administration method of CTX, the dosage and administration method of other drugs, as well as the characteristics of the included studies, and other relevant details, are detailed in Supplementary Table S4.

### 3.3 Quality of studies

**FIGURE 2:**
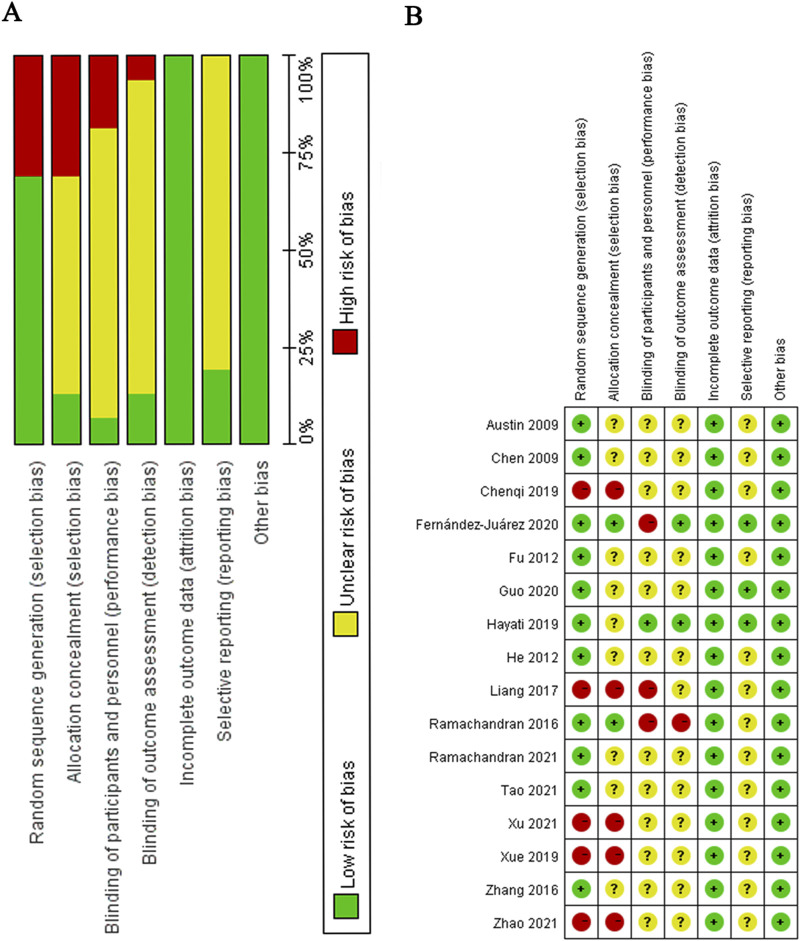
**(A)** Risk of bias graph **(B)** Risk of bias summary.

The quality of the sixteen included randomized controlled trials (RCT) is generally assessed as moderate (Figure 2), with the majority of studies offering minimal information regarding allocation concealment, participant and personnel blinding, and reporting biases. In the six retrospective clinical studies analyzed, those achieving scores between 7 and 9 were categorized as high-quality, those with scores ranging from 4 to 6 were regarded as moderate quality, and those scoring below 4 were classified as low-quality (Supplementary Table S5).

### 3.4 Complete remission and total remission rate

**FIGURE 3:**
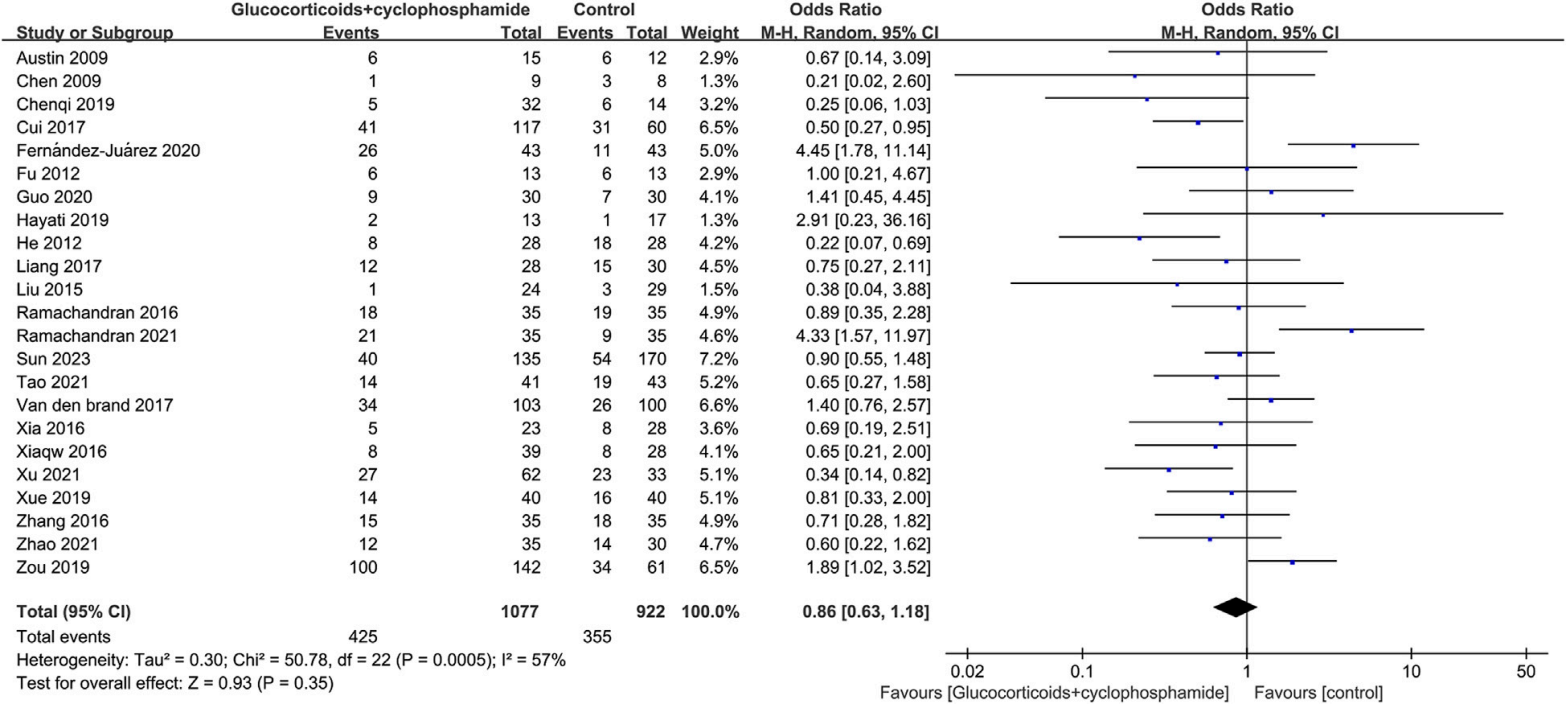
Forest plots of complete remission.

**FIGURE 4:**
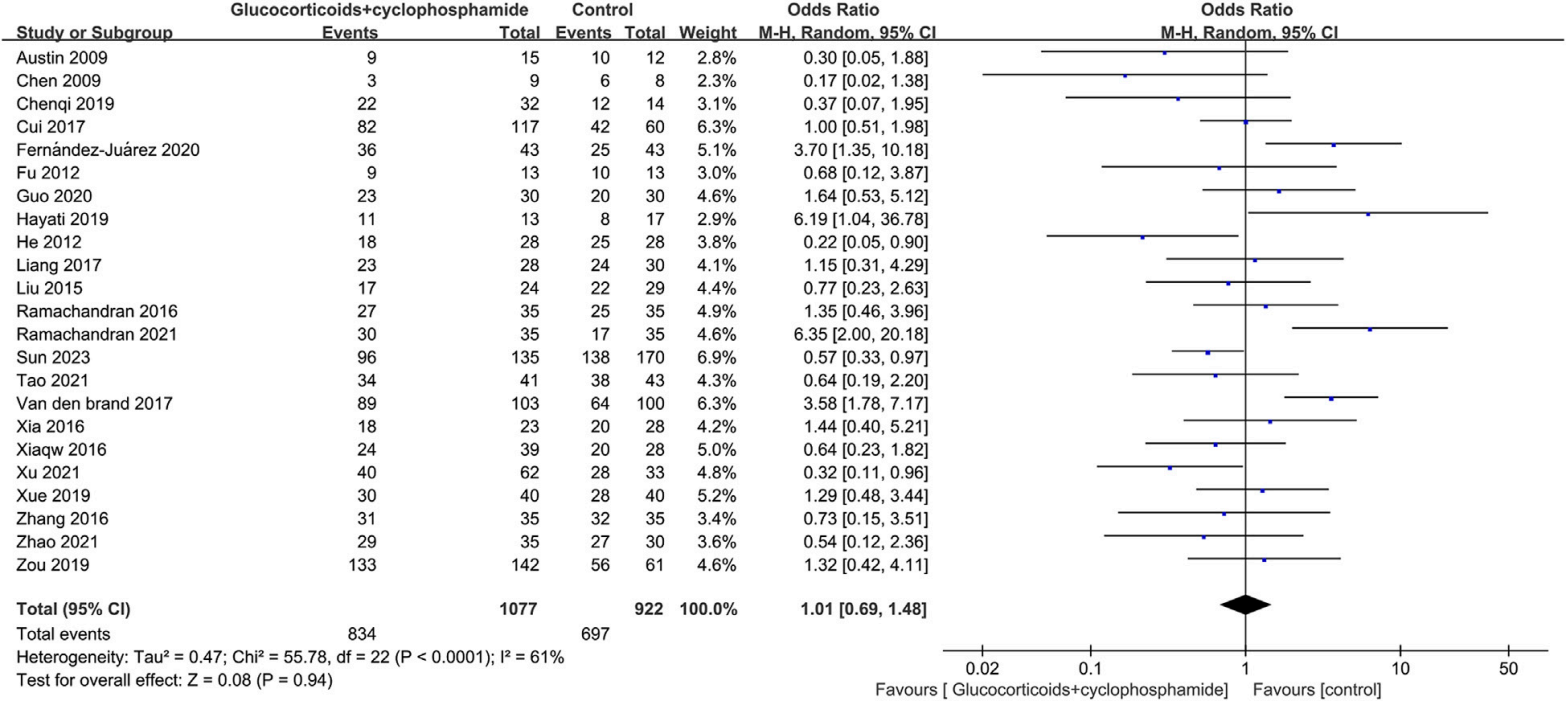
Forest plots of total remission.

The article discusses the rates of complete and overall remission in 23 studies involving 1,077 patients treated with GC combined with CTX. Additionally, 23 studies involving 894 patients in the control group (including GC combined with CsA, GC combined with TAC, GC combined with MMF, GC combined with LEF, TAC combined with RTX, GC combined with CsA and TAC, single use of TAC, single use of RTX, and single use of CsA) reported rates of complete and total remission. The complete and total remission rates for the GC combined with the CTX group were 39.46% and 77.44%, respectively, while the control group rates were 38.50% and 75.60%. No significant difference in remission rates was observed between the group receiving GC combined with CTX and the control group, leading to uncertainty regarding the superior efficacy of the two treatments for MN. CR: 23 studies, 1,077 patients, OR 0.86, 95% CI [0.63, 1.18], *p* = 0.35, *I* ^*2*^ = 57% (Figure 3). TR: 23 studies, 1,077 patients, OR 1.01, 95% CI [0.69, 1.48], *p* = 0.94, *I* ^*2*^ = 61% (Figure 4).

Subgroup analysis indicated that the CR in the GC combined with the CTX group was higher than that of the control group after a follow-up period of more than 12 months (OR 1.78, 95% CI [1.08, 2.92], *p* = 0.02, *I* ^*2*^ = 53%). Inter-study heterogeneity was reduced in retrospective clinical studies, Asian populations, SMN, and control groups for GC combined with MMF and TAC alone. (Table 1).

**TABLE 1:** Subgroup analysis of glucocorticoid combined with cyclophosphamide therapy on membranous nephropathy.

Subgroup		Complete remission				Total remission				Proteinuria			
		Study	OR [95%CI]	*p*-Value	*I* ^2^	Study	OR [95%CI]	*p*-Value	*I* ^2^	Study	SMD [95%CI]	*p*-Value	*I* ^2^
Total		23	0.86 [0.63, 1.18]	0.35	57%	23	1.01 [0.69, 1.48]	0.94	61%	15	0.10 [-0.09, 0.29]	0.30	56%
Study design													
RCT		16	0.83 [0.52, 1.31]	0.42	61%	16	0.95 [0.56, 1.62]	0.85	60%	11	0.05 [-0.15, 0.26]	0.59	42%
Retrospective		7	0.94 [0.62, 1.42]	0.77	47%	7	1.10 [0.62, 1.96]	0.74	67%	4	0.18 [-0.25, 0.61]	0.42	71%
Follow-up													
>12 months		7	1.78 [1.08, 2.92]	0.02	53%	7	2.14 [1.17, 3.92]	0.01	51%	4	−0.13 [-0.36, 0.11]	0.29	0%
≤12 months		16	0.63 [0.49, 0.80]	0.0002	0%	16	0.74 [0.53, 1.02]	0.07	24%	11	0.19 [-0.05, 0.43]	0.12	61%
Region													
Asia		20	0.76 [0.56, 1.04]	0.09	47%	20	0.89 [0.63, 1.27]	0.53	44%	14	0.12 [-0.09, 0.32]	0.27	58%
Europe		2	2.37 [0.77, 7.30]	0.13	76%	2	3.62 [2.04, 6.42]	<0.0001	0%	1	−0.06 [-0.48, 0.32]	0.79	NA
Population													
PMN		21	0.88 [0.62, 1.24]	0.45	60%	21	1.07 [0.72, 1.60]	0.73	62%	14	0.12 [-0.09, 0.32]	0.27	58%
SMN		2	0.66 [0.31, 1.41]	0.28	0%	2	0.50 [0.18, 1.41]	0.19	0%	1	−0.06 [-0.49, 0.37]	0.79	NA
Intervention													
GC	Oral	17	0.69 [0.51, 0.95]	0.02	35%	17	0.50 [0.18, 1.41]	0.19	0%	11	0.18 [-0.06, 0.43]	0.14	63%
	Oral + Intravenous	5	1.80 [0.90, 3.62]	0.10	65%	5	2.93 [1.74, 4.94]	<0.0001	26%	3	−0.12 [-0.39, 0.16]	0.40	0%
CTX	Oral	6	1.41 [0.68, 2.94]	0.36	78%	6	2.29 [1.25, 4.20]	0.007	60%	4	0.11 [-0.36, 0.57]	0.66	79%
	Intravenous	17	0.72 [0.53, 0.98]	0.04	31%	17	0.70 [0.50, 0.97]	0.03	18%	11	0.08 [-0.12, 0.28]	0.42	38%
Control													
GC + CsA		5	0.55 [0.34, 0.90]	0.02	0%	5	0.59 [0.33, 1.04]	0.07	0%	3	0.05 [-0.22, 0.33]	0.7	0%
GC + TAC		9	0.78 [0.42, 1.44]	0.43	70%	9	0.90 [0.49, 1.67]	0.74	58%	7	0.22 [-0.13, 0.58]	0.22	72%
GC + MMF		2	1.34 [0.36, 4.98]	0.66	0%	2	2.03 [0.23, 17.8]	0.52	67%	NA	NA	NA	NA
TAC		2	0.48 [0.17, 1.40]	0.18	35%	2	0.73 [0.24, 2.20]	0.58	10%	2	0.08 [-0.85, 1.10]	0.86	80%

Subgroup		Serum albumin				Serum creatinine			
		Study	SMD [95%CI]	*p*-Value	*I* ^2^	Study	SMD [95%CI]	*p*-Value	*I* ^2^
Total		14	−0.22 [−0.49, 0.06]	0.13	75%	11	−0.19 [−0.36, −0.01]	0.04	24%
Study design									
RCT		11	−0.24 [−0.59, 0.12]	0.19	81%	9	−0.19 [−0.41, 0.03]	0.09	39%
Retrospective		3	−0.20 [−0.50, 0.11]	0.21	0%	2	−0.18 [−0.54, 0.19]	0.34	0%
Follow-up									
>12 months		4	0.18 [−0.31, 0.66]	0.48	76%	4	−0.20 [−0.45, 0.05]	0.11	0%
≤12 months		10	−0.37 [−0.67, −0.08]	0.01	66%	7	−0.20 [−0.48, 0.09]	0.18	51%
Region									
Asia		13	−0.28 [−0.53, −0.03]	0.03	66%	10	−0.18 [−0.38, 0.02]	0.08	31%
Europe		1	0.71 [0.27, 1.14]	0.001	NA	1	−0.24 [−0.66, 0.18]	0.27	NA
Population									
PMN		13	−0.24 [−0.54, 0.06]	0.12	77%	10	−0.26 [−0.42, −0.09]	0.002	0%
SMN		1	−0.02 [−0.45, 0.41]	0.93	NA	1	0.34 [−0.09, 0.77]	0.13	NA
Intervention									
GC	Oral	10	−0.40 [0.−71, −0.10]	0.009	68%	7	−0.25 [−0.47, −0.03]	0.02	10%
	Oral + Intravenous	3	0.33 [0.0–9, 0.76]	0.13	57%	3	−0.27 [−0.54, 0.01]	0.05	0%
CTX	Oral	3	0.33 [−0.09, 0.76]	0.13	57%	3	−0.27 [−0.54, 0.01]	0.05	0%
	Intravenous	11	−0.36 [−0.64, −0.08]	0.01	67%	8	−0.16 [−0.41, 0.10]	0.22	44%
Control									
GC + CsA		3	−0.37 [−0.86, 0.12]	0.13	67%	3	−0.33 [−0.61, −0.05]	0.02	0%
GC + TAC		6	−0.48 [−0.96, 0.00]	0.05	78%	4	−0.06 [−0.41, 0.29]	0.74	41%
GC + MMF		NA	NA	NA	NA	1	0.03 [−0.74, 0.80]	0.94	NA
TAC		2	−0.19 [−0.59, 0.21]	0.35	0%	1	−0.74 [−1.39, −0.10]	0.02	NA

RCT, randomize controlled trials; RTX, rituximab; PMN, primary membranous nephropathy; SMN, secondary membranous nephropathy; CR, complete remission; TR, total remission; GC, glucocorticosteroid; CsA, cyclosporine; TAC, tacrolimus; MMF, mycophenolate mofetil; LEF, leflunomide; CTX, cyclophosphamide; OR, odds ratio; SMD, standardized mean difference.

Subgroup analysis of the TR demonstrated that the combination of GC and CTX achieved a higher overall remission rate compared to the control group after a follow-up period of over 12 months (OR 2.14, 95% CI [1.17, 3.92], *p* = 0.01, *I* ^*2*^ = 51%). The TR of GC combined with CTX is higher than the control in the European region (OR 3.62, 95% CI [2.04, 6.42], *p* < 0.0001, *I* ^*2*^ = 0%). The TR of GC was higher than that of the control group when GC was administered both orally and intravenously (OR 2.93, 95% CI [1.74, 4.94], *p* < 0.0001, *I* ^*2*^ = 26%). The TR was higher in CTX administration than in the control group when the mode of administration was oral (OR 2.29, 95% CI [1.25, 4.20], *p* = 0.007, *I* ^*2*^ = 60%). SMN, control group for GC combined with CsA and reduced between-study heterogeneity with TAC alone. (Table 1).

### 3.5 Changes in proteinuria, serum albumin, and serum creatinine

**FIGURE 5:**
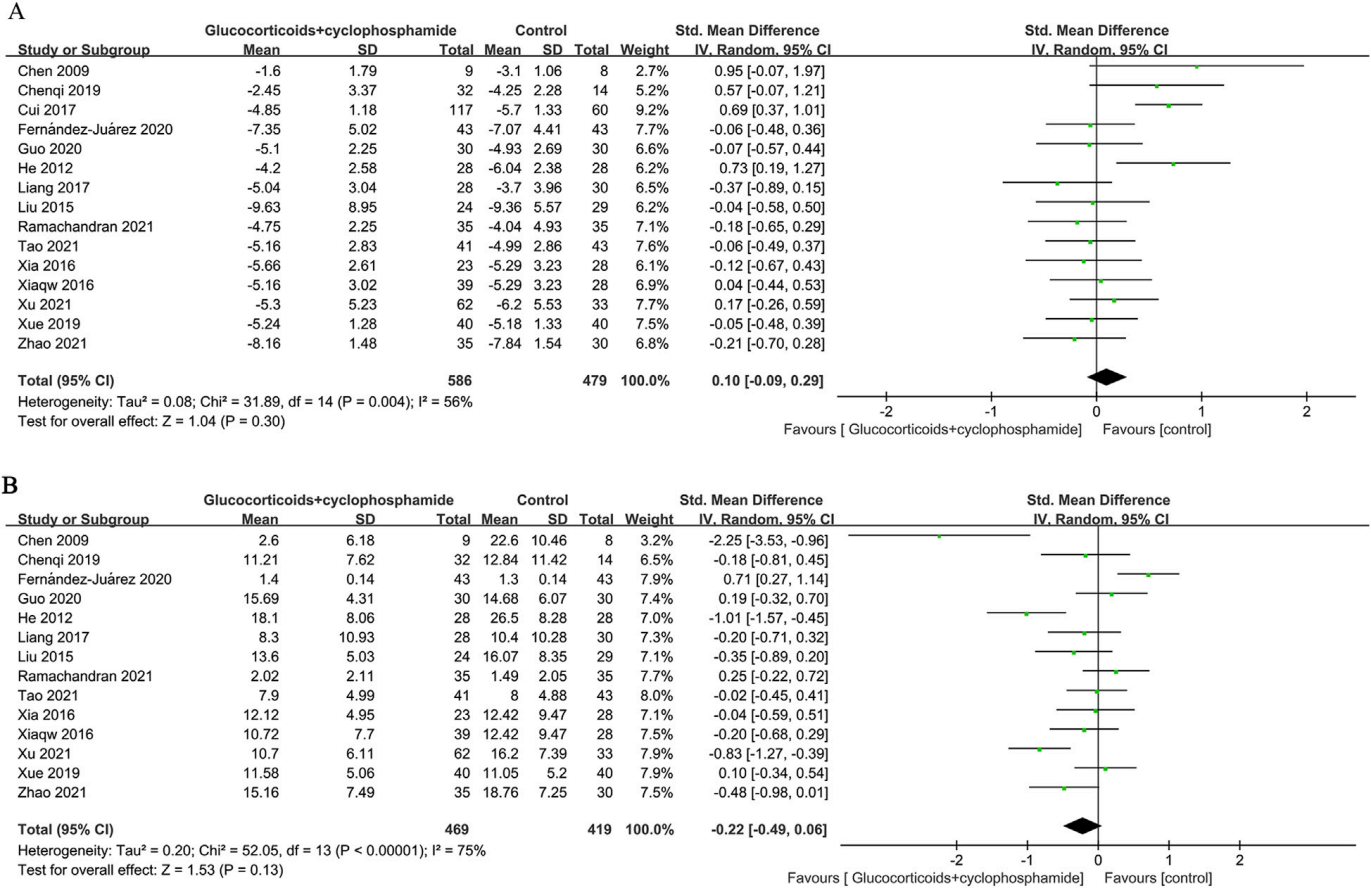
Forest plots of **(A)** proteinuria (g/24 h) and **(B)** serum albumin.

**FIGURE 6:**
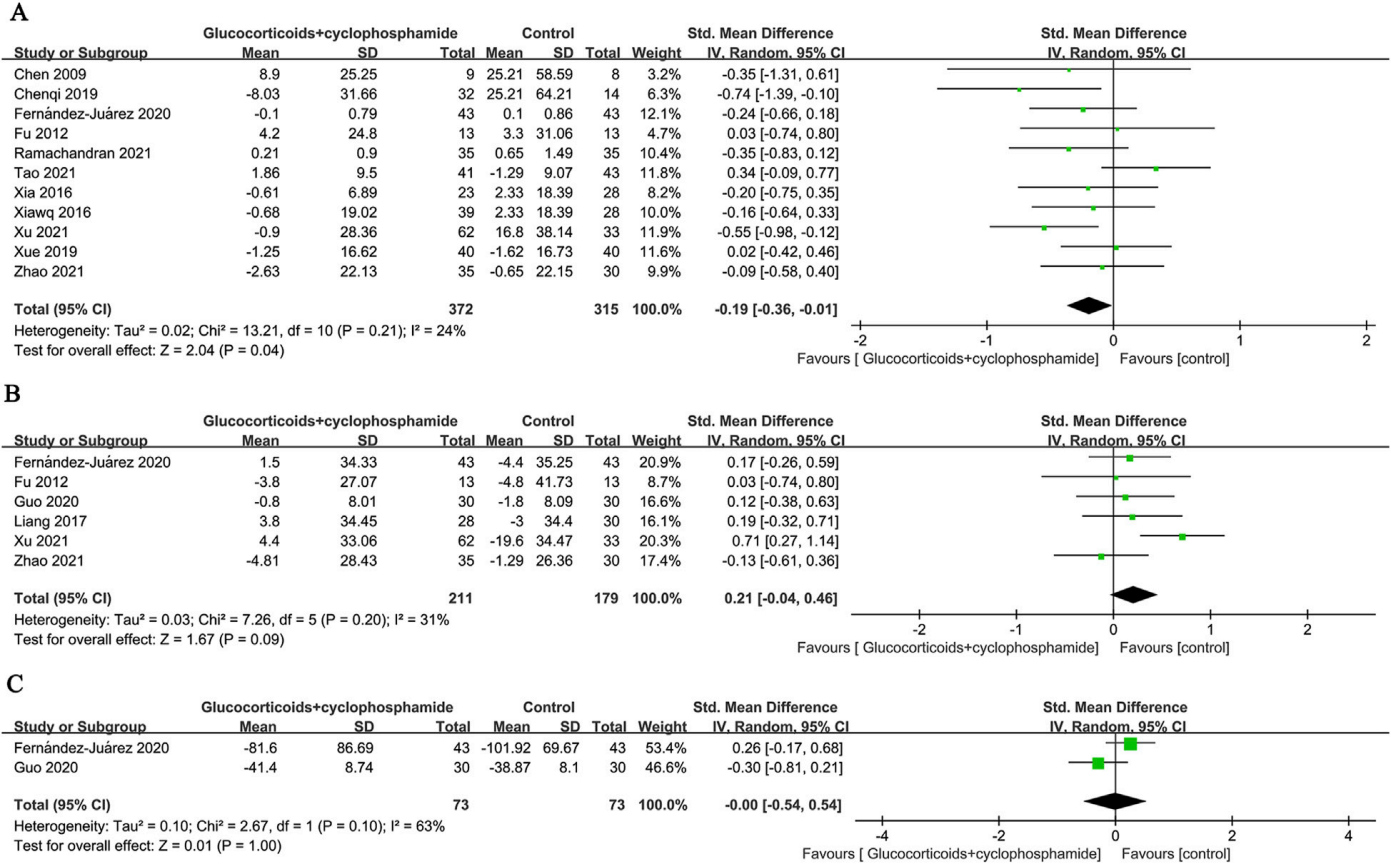
Forest plots of **(A)** serum creatinine, **(B)** eGFR, and **(C)** Anti-PLA2R.

Fifteen studies (586 patients) evaluated changes in 24-h urinary protein, 14 studies (469 patients) assessed serum albumin, and 11 studies (372 patients) examined serum creatinine before and after the intervention in the GC combined with the CTX group. There was no significant difference in the effectiveness of the GC combined with the CTX group in lowering urinary protein and increasing serum albumin compared with the control group (proteinuria: SMD 0.10, 95% CI [−0.09, 0.29], *p* = 0.3 Figure 5A; serum albumin: SMD −0.22, 95% CI [−0.49, 0.06], *p* = 0.13 Figure 5B). Heterogeneity was found between studies (Proteinuria: *I* ^*2*^ = 56%, Serum albumin: *I* ^*2*^ = 75%). However, the GC combined with the CTX group was more effective than the control group in reducing serum creatinine (SMD −0.19, 95% CI [−0.36, −0.01], *p* = 0.04, *I* ^*2*^ = 24% Figure 6A), and no significant heterogeneity was found between the studies.

Subgroup analysis showed that for PMN, the GC combined with the CTX group was more effective in lowering serum creatinine (SMD - 0.26, 95% CI [−0.42, −0.09], *p* = 0.002, *I* ^*2*^ = 0%, Table 1). When the control group received GC combined with CsA, the GC combined with the CTX group was more effective in reducing serum creatinine. (SMD - 0.33, 95% CI [−0.61, −0.05], *p* = 0.02, *I* ^*2*^ = 0%, Table 1).

### 3.6 Changes in eGFR and Anti-PLA2R

Six (211 patients) and two (73 patients) studies evaluated changes in eGFR and Anti-PLA2R before and after intervention in the GC combined with the CTX group, respectively. The results indicated no significant differences compared to the control group (eGFR: SMD 0.21, 95% CI [-0.04, 0.46], *p* = 0.09, *I* ^*2*^ = 31% Figure 6B; Anti-PLA2R: SMD-0.00, 95% CI [−0.54, 0.54], *p* = 1.00, *I* ^*2*^ = 63% Figure 6C).

### 3.7 Relapse rate

**FIGURE 7:**
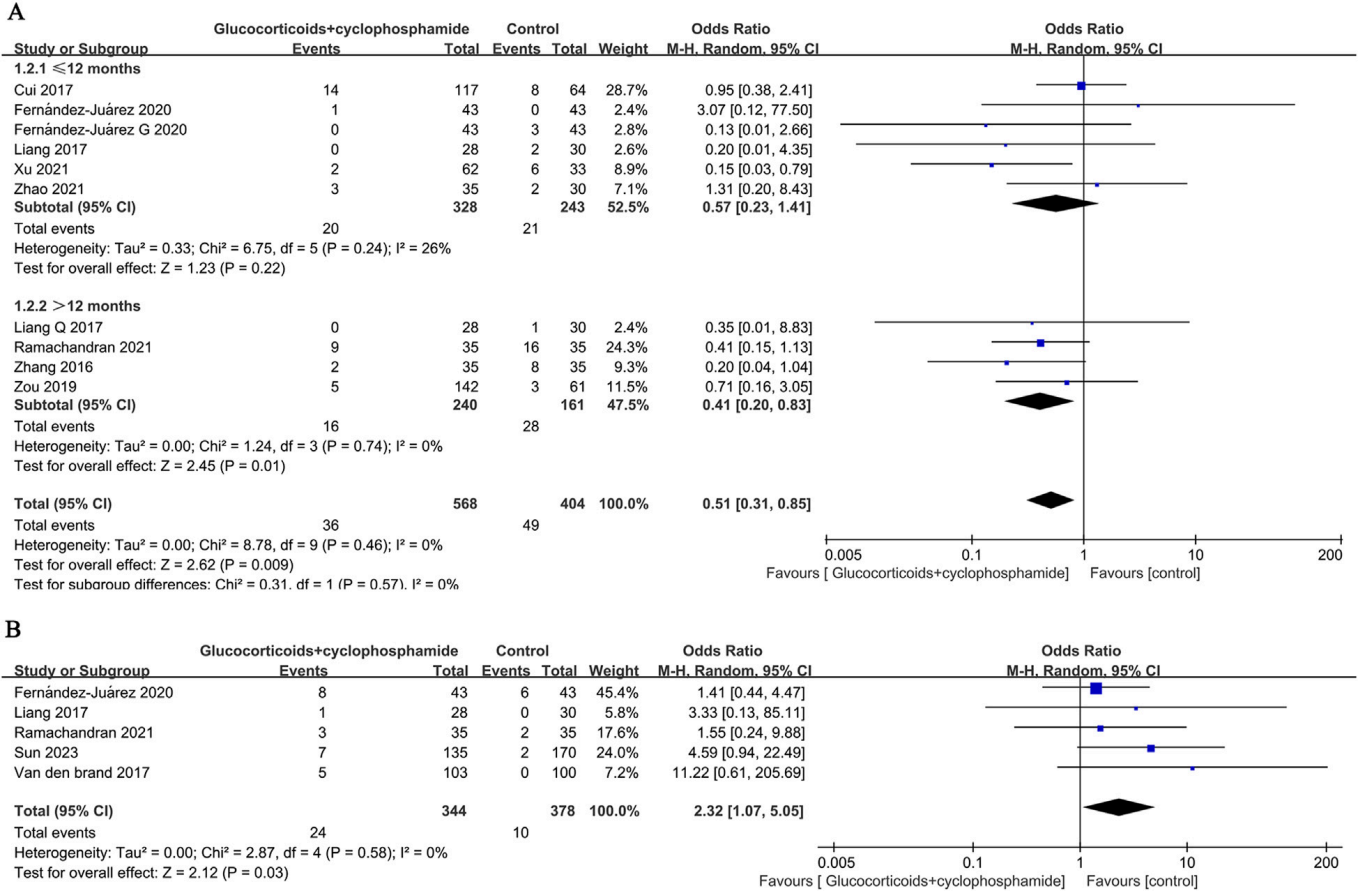
Forest plot of **(A)** recurrence rates at ≤ 12 months and >12 months and **(B)** serious adverse events.

The recurrence rates of GC combined with CTX and control groups were reported in 8 papers (569 patients), and 8 papers (404 patients), respectively (6.3% vs. 12.1%, OR 0.51, 95% CI [0.31, 0.85], *p* = 0.009, *I* ^*2*^ = 0%). Because the time to recurrence of patients reported in the literature did not coincide with the time to follow-up, the eight papers were categorized into 10 studies according to the time to recurrence, assessment of time to recurrence ≤12 months (OR 0.57, 95% CI [0.23, 1.41], *p* = 0.22, *I* ^*2*^ = 26%), >12 months (OR 0.41, 95% CI [0.20, 0.83], *p* = 0.01, *I* ^*2*^ = 0%) (Figure 7A). The results indicate that the relapse rate of MN treated with GC combined with CTX is lower than that of the control group, particularly concerning the long-term relapse rate. There is no significant heterogeneity among the studies.

### 3.8 Adverse events

The GC combined with CTX group had a significantly higher risk of alopecia (OR 2.82, 95% CI [1.24, 6.44], *p* = 0.01, *I* ^*2*^ = 0%), leukopenia (OR 4.88, 95% CI [2.15, 11.10], *p* = 0.0002, *I* ^*2*^ = 0%), and liver damage (OR 2.77, 95% CI [1.33, 5.77], *p* = 0.006, *I* ^*2*^ = 0%) compared to the control group (Table 2). The occurrence risk of infection (OR 1.37, 95% CI [0.77, 2.44], *p* = 0.28, *I* ^*2*^ = 46%), pneumonia (OR 0.84, 95% CI [0.30, 2.36], *p* = 0.75, *I* ^*2*^ = 4%), glucose intolerance (OR 0.67, 95% CI [0.24, 1.87], *p* = 0.44, *I* ^*2*^ = 19%), new-onset hypertension (OR 0.61, 95% CI [0.25, 1.50], *p* = 0.28, *I* ^*2*^ = 37%), worsening hypertension (OR 0.72, 95% CI [0.24, 2.22], *p* = 0.57, *I* ^*2*^ = 0%), gastrointestinal intolerance (OR 1.35, 95% CI [0.63, 2.89], *p* = 0.45, *I* ^*2*^ = 32%), tremor (OR 0.38, 95% CI [0.05, 2.76], *p* = 0.34, *I* ^*2*^ = 58%), hyperglycemia (OR 0.77, 95% CI [0.47, 1.24], *p* = 0.28, *I* ^*2*^ = 0%), zoster or skin eruption (OR 3.80, 95% CI [0.84, 17.14], *p* = 0.08, *I* ^*2*^ = 19%), venous thrombosis (OR 1.25, 95% CI [0.48, 3.22], *p* = 0.65, *I* ^*2*^ = 0%) did not show significant differences between the GC combined with CTX group and the control group (Table 2). There was a statistical difference in the total non-serious adverse events (OR 1.30, 95% CI [1.01, 1.67], *p* = 0.04, *I* ^*2*^ = 32%) between the two groups (Table 2).

**TABLE 2:** Meta-analysis of non-serious adverse events in MN patients treated with GC + CTX group and control group.

Non-serious adverse events						
	Study	GC + CTX group n/N	Control group n/N	OR [95%CI]	*p*-value	*I* ^2^
Total	18	359/3905	268/3776	1.30 [1.01, 1.67]	0.04	32%
Alopecia	5	22/249	7/278	2.82 [1.24, 6.44]	0.01	0%
Infection	11	111/551	74/501	1.37 [0.77, 2.44]	0.28	46%
Pneumonia	5	9/106	10/104	0.84 [0.30, 2.36]	0.75	4%
Leukopenia	8	34/346	5/379	4.88 [2.15, 11.10]	0.0002	0%
Liver damage	8	26/333	9/373	2.77 [1.33, 5.77]	0.006	0%
Glucose intolerance	4	21/216	19/161	0.67 [0.24, 1.87]	0.44	19%
New-onset hypertension	6	17/315	33/354	0.61 [0.25, 1.50]	0.28	37%
Worsening hypertension	2	6/70	8/70	0.72 [0.24, 2.22]	0.57	0%
Gastrointestinal intolerance	12	40/465	33/379	1.35 [0.63, 2.89]	0.45	32%
Tremor	4	7/148	15/143	0.38 [0.05, 2.76]	0.34	58%
Hyperglycemia	10	42/530	45/472	0.77 [0.47, 1.24]	0.28	0%
Zoster/Skin eruption	5	13/256	2/288	3.80 [0.84, 17.14]	0.08	19%
Venous thrombosis	3	11/320	8/274	1.25 [0.48, 3.22]	0.65	0%

n, number of patients experiencing non-serious adverse events; N, total number of patients; GC, glucocorticosteroid; CTX, cyclophosphamide; OR, odds ratio.

Five studies (722 patients) were included to evaluate the differences between the GC combined with CTX group and the control group in terms of serious adverse reaction events (OR 2.32, 95% CI [1.07, 5.05], *p* = 0.03, *I* ^*2*^ = 0% Figure 7B). The results showed that compared to the control group, the GC combined with the CTX group had a higher incidence of serious adverse reactions in treating MN.

### 3.9 Publication bias and sensitivity analysis

**FIGURE 8:**
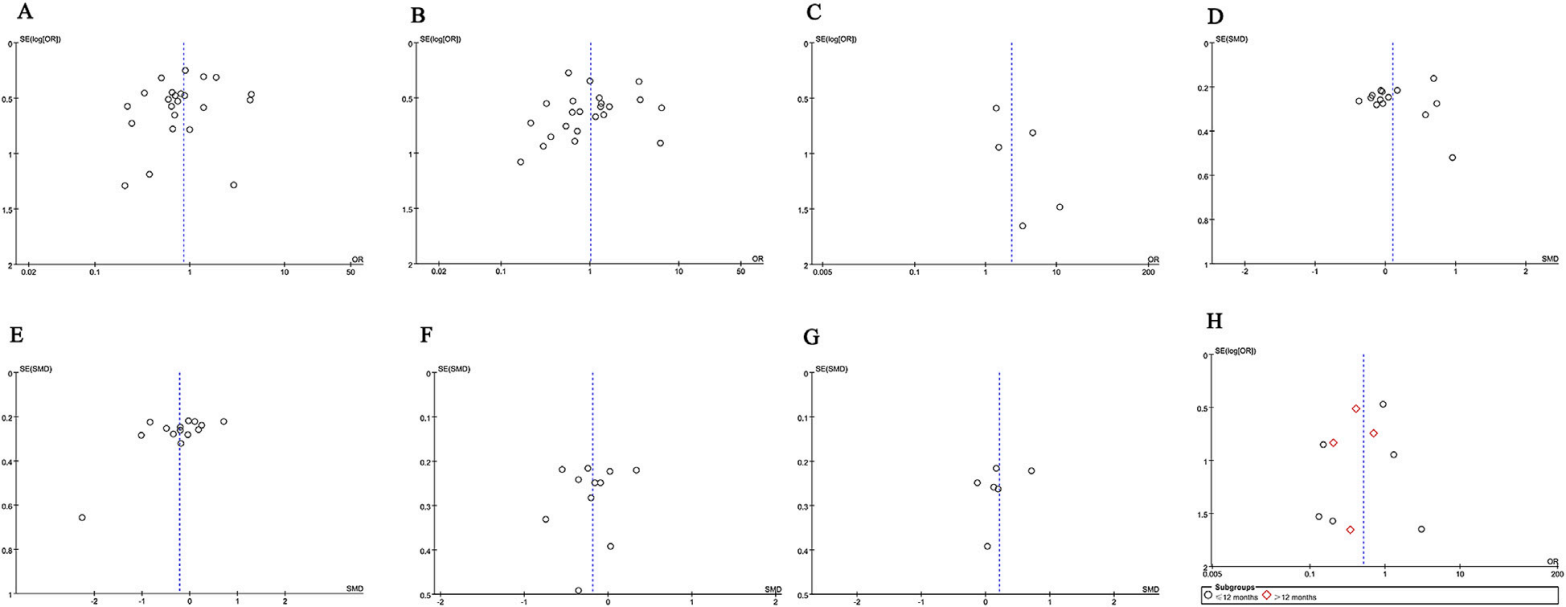
Funnel plots of **(A)** CR, **(B)** TR, **(C)** serious adverse events, **(D)** proteinuria (g/24 h), **(E)** serum albumin, **(F)** serum creatinine, **(G)** eGFR, and **(H)** recurrence rates at ≤ 12 months and >12 months.

We used Egger’s test and funnel plots to assess publication bias. The *p*-values for Egger’s test of each indicator: CR (*p* = 0.351), TR (*p* = 0.539), serious adverse events (*p* = 0.241), proteinuria (*p* = 0.762), serum albumin (*p* = 0.052), serum creatinine (*p* = 0.555), eGFR (*p* = 0.454) and relapse rate (*p* = 0.503). Egger’s test showed no publication bias was observed. The symmetry of the funnel plot is acceptable (CR (Figure 8A), TR (Figure 8B), serious adverse events (Figure 8C), proteinuria (Figure 8D), serum albumin (Figure 8E), serum creatinine (Figure 8F), eGFR (Figure 8G), and relapse rate (Figure 8H).

**FIGURE 9:**
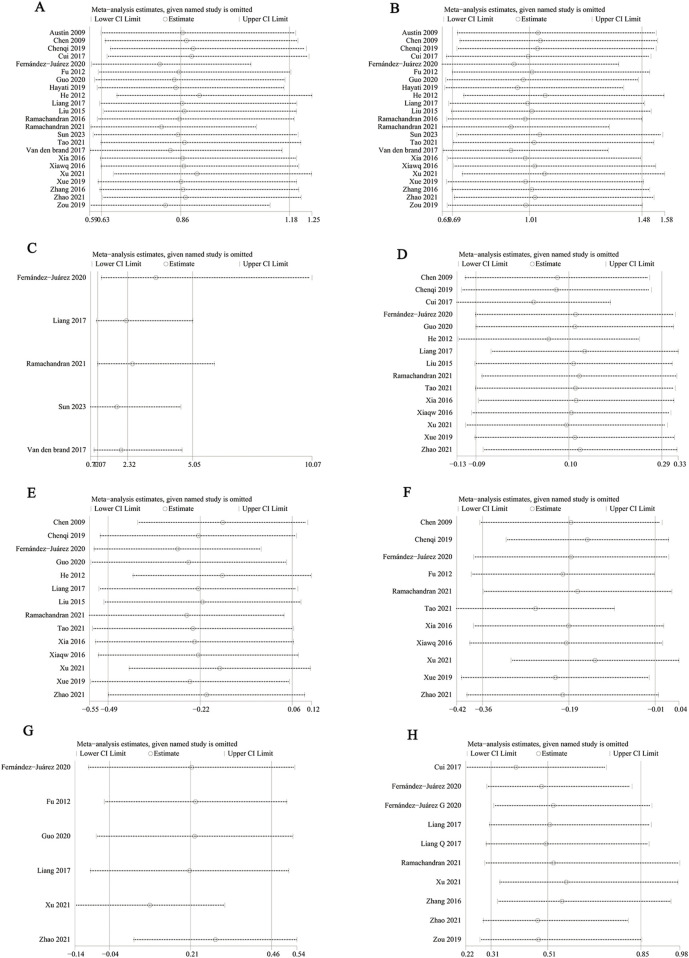
Sensitivity analysis of **(A)** CR, **(B)** TR, **(C)** serious adverse events, **(D)** proteinuria (g/24 h), **(E)** serum albumin, **(F)** serum creatinine, **(G)** eGFR, and **(H)** relapse rate.

We performed a one-way sensitivity analysis of CR (Figure 9A), TR (Figure 9B), serious adverse events (Figure 9C), proteinuria (Figure 9D), serum albumin (Figure 9E), serum creatinine (Figure 9F), eGFR (Figure 9G), and relapse rate (Figure 9H). The effect of each study on OR or SMD in the GC combined with the CTX group was assessed by excluding the individual studies one by one. Sensitivity analyses showed that the new *p* values remained statistically insignificant after the exclusion of CR, TR, relapse rate, and proteinuria from any individual study.

After excluding data from studies reported by Sun et al. (2023), van den Brand et al. (2017), the *p*-values for serious adverse events were (*p* = 0.17) and (*p* = 0.36), which were not statistically significant, these articles made the data of serious adverse events unstable.

When we excluded the Serum albumin data reported in this study by Fernández-Juárez (2020), it had a *p*-value <0.05, and the heterogeneity decreased (SMD −0.28, 95% CI [−0.53, −0.03], *p* = 0.03, *I* ^*2*^ = 66%), suggesting that this article led to instability in the results and was a source of heterogeneity.

After excluding data from the studies by Weizhen et al. (2009), Qi et al. (2019), Fernández-Juárez et al. (2020), Ramachandran et al. (2021), Weiqing et al. (2016), and Lili et al. (2021), the *p*-values for serum creatinine were (*p* = 0.06), (*p* = 0.08), (*p* = 0.08), (*p* = 0.09), (*p* = 0.06), (*p* = 0.06), (*p* = 0.14), respectively, which were not statistically significant. These articles contributed to instability in the GC combined with the CTX group regarding the reduction of serum creatinine.

However, when we excluded the data reported by Zhao et al. (2021) (Bianjie and Shengkai, 2021), the *p*-value for eGFR was less than 0.05, and the heterogeneity decreased (SMD 0.29, 95% CI [0.04, 0.54], *p* = 0.02, *I* ^*2*^ = 18%), suggesting that this study contributed to result instability and was a source of heterogeneity.

## 4 Discussion

Numerous evidence-based studies indicate that patients with IMN presenting as nephrotic syndrome do not respond adequately to corticosteroid treatment alone and necessitate the addition of immunosuppressants to enhance efficacy (Hogan et al., 1995; Perna et al., 2004). Currently, the immunosuppressants commonly used in combination with GC in clinical practice include CTX, CsA, and TAC. The standard treatment regimen for CTX involves the concurrent administration of sufficient doses of GC and CTX, followed by a gradual reduction in GC dosage after 8–12 weeks. The Ponticelli regimen is frequently utilized for IMN treatment (Chinese Expert Group on Immunosuppressive Therapy for Adult Nephrotic Syndrome, 2014) (Methylprednisolone 0.5–1.0 g/d Intravenous shock for 3 d and then switched to oral prednisone 0.5 mg/kg/day × 27 d alternating with oral CTX 2.0–2.5 mg/kg/day × 30 days for 3 treatments for a total course of 6 months, with a total of up to 11 g of hormones and a cumulative amount of CTX of 10 g or more). China often adopts the modified Ponticelli regimen (Yan et al., 2012) (Methylprednisolone 0.5 g/day intravenous shock for 3 days, oral prednisone 0.5 mg/kg/day × 27 days, CTX was changed to 0.75 g/m^2^ shock treatment every 2, 4, 6 months, discontinue prednisone, the total course of 6 months). In about 70% of patients, CNIs can induce remission of nephrotic syndrome (Cattran et al., 2001; Praga et al., 2007). MMF, an inhibitor of inosine monophosphate dehydrogenase, can selectively inhibit the proliferation of B and T lymphocytes, exhibiting strong immunosuppressive effects. LEF, an immunomodulator, acts by inhibiting the mitochondrial enzyme known as dihydroorotate dehydrogenase (Chen et al., 2014). RTX, a monoclonal antibody that targets the CD20 antigen present in B lymphocytes, may serve as an effective and safe alternative therapy when combined with steroids and alkylating agents (van den Brand et al., 2017).

In this study, we conducted a systematic evaluation of the efficacy and safety of GC combined with CTX in patients diagnosed with MN. Our meta-analysis revealed that GC combined with CTX was more effective in reducing serum creatinine compared to the control group, and it had a lower long-term recurrence rate. However, the safety profile of GC combined with CTX in treating MN was poor. Our meta-analysis indicated a significantly higher risk of hair loss, leukopenia, and liver damage in the GC combined with the CTX group compared to the control group. Conversely, there were no significant differences observed in the incidence of infection, pneumonia, glucose intolerance, new-onset hypertension, worsening hypertension, gastrointestinal intolerance, tremor, hyperglycemia, zoster or skin eruption, and venous thrombosis between the two groups. Notwithstanding, a notable disparity in the total number of non-serious adverse events existed between the groups. The dropout rate due to adverse reactions was reported in only one study, thus precluding its inclusion in the meta-analysis. The included studies predominantly highlighted severe adverse events such as infections, pneumonia, sepsis-inducing respiratory infections, cancer, avascular necrosis of the femoral head, severe acute kidney injury, and mortality. Given the scarcity of studies explicitly discussing severe adverse events, a meta-analysis specifically focusing on severe adverse events was conducted. The outcomes revealed a higher risk of serious adverse reactions in the GC combined with the CTX group compared to the control group. However, sensitivity analysis detected significant instability in serious adverse reaction events, suggesting insufficient evidence to definitively establish that the safety profile of GC combined with CTX in managing MN is inferior to that of the control group.

Subgroup analysis revealed that GC combined with CTX significantly improved both the TR and CR following over 12 months of treatment. The GC administration involved both oral and intravenous routes, specifically alternating GC and CTX (Ponticelli regimen or modified Ponticelli regimen), resulting in a higher TR compared to the control group. This finding aligns with studies by Fernández-Juárez et al. (2020), Ramachandran et al. (2021), and Xia (Weiqing et al., 2016), which demonstrated that alternating CTX and GC markedly improved the number of patients achieving remission in the treatment of MN, especially in terms of long-term remission. A randomized controlled trial carried out by Chen et al. (2010) compared GC combined with TAC to the classic GC combined with CTX. Research indicated that at 6 months, the remission rates in the TAC group were markedly elevated compared to those in the CTX group, patients in the TAC group saw a substantial improvement in reduced urinary protein and increased serum albumin. Xu et al. (Lili et al., 2021) demonstrated that the combination of GC and CsA had a faster onset of action in the treatment of IMN compared to GC and CTX, with a comparable overall treatment effect. Qiu et al. (2017) meta-analysis showed that CNIs significantly increased the TR, improved serum albumin levels, and decreased proteinuria after 6 months of treatment. Our study indicated that the combination of GC and CTX significantly increased both the TR and CR after more than 12 months of treatment, outperforming other drugs in the control group, but did not show a significant advantage in decreasing urinary protein or improving serum albumin. Ramachandran et al study demonstrated that after 12 months of treatment, patients receiving TAC therapy exhibited a significant decrease in eGFR and a significant increase in serum creatinine (Ramachandran et al., 2016). Qiu et al. (2017) meta-analysis found no notable difference in serum creatinine between CNIs and CTX. However, our study indicated that GC combined with CTX was more effective in reducing serum creatinine compared to the control group’s drugs. This discrepancy in results may be attributed to new research evidence, the inclusion of other drugs besides CNIs, and differences in sample sizes. Liang et al. (2017) study did not observe an increase in serum creatinine or a decrease in eGFR during TAC treatment. Our meta-analysis did not conduct a subgroup analysis due to the limited literature on eGFR. From the existing data analysis, it cannot be concluded that GC combined with CTX is less effective than other drugs in reducing eGFR. The analysis of Anti-PLA2R was based on only two studies, which showed no statistical difference between the groups, highlighting the need for additional data.

One of the primary drawbacks of CNIs is the significant relapse rate following their discontinuation, with 40%–60% of patients facing a recurrence of symptoms. Numerous studies indicate a tendency for MN to relapse after discontinuation or gradual reduction of CNIs, with a relapse rate of 13%–50% (Chen et al., 2010; Ramachandran et al., 2016). In the study by Praga et al. (2007), the relapse rate after discontinuation was 47%, which did not differ from the placebo group. Our meta-analysis revealed that the combination of GC and CTX treatment results in a lower recurrence rate of MN compared to the control group, with a particularly significant reduction in long-term recurrence rates beyond 12 months of treatment. Previous research has demonstrated that prolonged use of CTX heightens the risk of infections, which can result in severe adverse effects including leukopenia and hair loss (Ahlmann and Hempel, 2016; Eriguchi et al., 2009), this finding aligns with the results of our meta-analysis. However, determining whether it is higher than any individual drug in the control group requires more data. The incidence of elevated serum creatinine is lower in the GC combined with the CTX group than in the control group. Existing meta-analyses have shown that TAC and CTX do not differ significantly in other adverse reactions (Zhu et al., 2017). Another meta-analysis also found no substantial difference in the overall rate of adverse drug reactions between the CNIs group and the CTX group (Qiu et al., 2017).

In addition to the previously mentioned subgroup analyses, other subgroup analysis results indicate that for PMN, the combination of GC and CTX significantly reduces serum creatinine levels. However, this finding might be influenced by the limited data from our meta-analysis, which included only two studies on non-primary membranous nephropathy. Oral administration of CTX has demonstrated a higher overall remission rate compared to the control group. Notably, there is ongoing discussion regarding the use of intravenous cyclophosphamide CTX in treating PMN. The “2012 KDIGO Guidelines” recommend only oral CTX, while studies by Dede et al. (2008), Yuan et al. (2011) have shown that intravenous cyclophosphamide CTX can enhance the remission rate in PMN patients. Contrarily, some studies do not support this conclusion (Falk et al., 1992; Branten et al., 1998). The findings from our meta-analysis indicate that TR is more effective when CTX is given orally compared to the control group; however, this outcome might be affected by the small number of studies within each subgroup and the reduced size of the patient populations. This restriction leads to a lack of sufficient data support, preventing a definitive conclusion. When combined with CsA, the combination of GC and CTX is superior to the control group in reducing serum creatinine. According to the research by Zhang et al. (Min, 2016), while the CTX group experienced a delayed onset of remission compared to the CsA group, the overall treatment outcomes were not significantly different between the two groups after 12 months. Additionally, at the 24-month mark, the recurrence rate in the CTX group was found to be lower than in the CsA group.

CTX is a non-specific, periodic medication that disrupts the normal functioning of DNA and RNA. This interference impedes cell proliferation and suppresses the immune system by damaging susceptible lymphocytes. It induces systemic destruction of leukocytes and mature plasma cells. Earlier research has shown that alkylating agents are effective in managing the most severe forms of PMN (Howman et al., 2013; van de Logt et al., 2018; Oleinika et al., 2019). However, long-term use of CTX can lead to serious adverse events. Serious adverse reactions associated with CTX typically require cumulative doses to exert immunosuppressive effects. This may explain the higher TR and CR observed with the combination of GC and CTX in the treatment of MN over more than 12 months, though this often comes with relatively severe adverse reactions.

TAC’s mechanism of action involves disrupting calcium-dependent signaling pathways, which ultimately inhibits the transcription of genes such as IL-2R, IL-2, and IFN-γ, thereby suppressing T cell proliferation. Additionally, it inhibits early lymphocyte aggregation during immune responses and prevents aggregated lymphocytes from attracting other inflammatory cells. This dual inhibitory effect enables TAC to be used not only to prevent immune responses but also to treat established immune responses and autoimmune diseases (Shaw et al., 1995; Undre et al., 1999). Moreover, TAC boosts the immunosuppressive action of glucocorticoids by elevating the affinity of glucocorticoid receptors (Ning and Sánchez, 1993). Studies show that the remission rate for GC combined with TAC is considerably higher in the initial 3 months compared to GC combined with CTX (Zou et al., 2019). The main mechanism of action for both CsA and TAC is comparable, as they both work to inhibit T cell activation and the proliferation of T cell-dependent B cells. Additionally, research has shown that TAC treatment is linked to an increased risk of recurrence, infections, tumors, nephrotoxicity, and neurotoxicity (He et al., 2012; Ramachandran et al., 2016; Hoxha et al., 2015; Zhu et al., 2018). This further emphasizes that CNIs may achieve a higher remission rate in the treatment of early-stage MN. Reports suggest that RTX could serve as an alternative to CTX as the main immunosuppressive therapy for patients with PMN and nephrotic syndrome (Howman et al., 2013). However, it is important to note that rituximab has a substantial non-response rate and a low partial remission rate in this patient population (van den Brand et al., 2017; Dahan et al., 2017). Chen et al.'s meta-analysis reported that RTX treatment for IMN is superior to other immunosuppressants (Chen et al., 2021). An observational study found that the gradual discontinuation of CsA or TAC while using RTX can reduce the recurrence rate (Segarra et al., 2009). Moreover, studies indicate that MMF is anticipated to emerge as the first-line treatment for IMN (Peng et al., 2012), and the combination of GC and LEF for the treatment of PLA2R-associated PMN is also a safe and effective method (Guo et al., 2020).

During literature screening, no relevant literature was found on primary membranous nephropathy in kidney transplant recipients. However, the recurrence rate of idiopathic membranous nephropathy (IMN) after kidney transplantation ranges from 30% to 50%, warranting attention. Phospholipase A2 Receptor (PLA2R) antibodies are significant in the recurrence of IMN, and RTX can reduce these antibodies, leading to decreased proteinuria levels in IMN patients. Therefore, utilizing RTX to manage the recurrence of membranous nephropathy post-kidney transplantation proves to be an effective treatment. A study revealed that treatment with RTX for recurrent IMN resulted in 50% of patients achieving complete remission during the 12-month follow-up (Fervenza et al., 2010).

Although this study included a relatively large number of cases, there are still various confounding factors. Some randomized controlled trials had limited sample sizes and brief follow-up durations, while others exhibited significant heterogeneity in study outcomes and unstable data, potentially leading to bias. Detailed individual patient data were not available, resulting in potential heterogeneity in baseline characteristics. Additionally, the majority of the included patients were Asian, so the effectiveness and safety of GC combined with CTX in other regions cannot be conclusively determined. Data on the treatment of SMN patients is limited, as is the information regarding the efficacy of using glucocorticoids alone or other single drugs for MN treatment, preventing any definitive conclusions from being drawn.

## 5 Conclusion

Based on the above, current evidence suggests that the combination of GC and CTX has better long-term efficacy and lower recurrence rates compared to other drugs used in the treatment of MN. Additionally, it is considered the optimal drug combination for normalizing serum creatinine levels. Although many studies propose alternative drugs to replace CTX to avoid its severe adverse reactions, the long-term safety of other immunosuppressants remains inconclusive. This is especially applicable to PMN patients who fail to achieve remission within 12 months. Furthermore, there is a lack of data regarding the treatment of SMN patients and the effectiveness of using GC alone or other drugs alone for the treatment of MN, which is not reported in this paper. The possibility of replacing CTX is still uncertain, and there is a need for more large-scale prospective studies to gather additional data for validation.

## Data Availability

The original contributions presented in the study are included in the article/Supplementary Material, further inquiries can be directed to the corresponding authors.
