# Supplementary material for: Efficacy and safety of glucocorticoid combined with cyclophosphamide therapy on membranous nephropathy: a systematic review and meta-analysis
Source: Front Pharmacol. 2024 Nov 27;15:1480638. doi: 10.3389/fphar.2024.1480638 (PMC11631627; doi:10.3389/fphar.2024.1480638)
Supplement: Supplementary file 2 [file Table2.DOCX]

Supplementary Material

**Supplementary Table S2** Literature search strategy

| **PubMed** |
| --- |
| ((("Cyclophosphamide"[Mesh]) OR (((((((((((((Sendoxan) OR (B 518)) OR (Cyclophosphamide Anhydrous)) OR (Cytophosphane)) OR (Cyclophosphamide Monohydrate)) OR (Cytophosphan)) OR (Cytoxan)) OR (Endoxan)) OR (Neosar)) OR (NSC 26271)) OR (Procytox)) OR (Monohydrate)) OR (Cyclophosphane))) AND (("Glucocorticoids"[Mesh]) OR (((Glucocorticoid) OR (Glucocorticoid Effect)) OR (Glucorticoid Effects)))) AND (("Glomerulonephritis, Membranous"[Mesh]) OR ((((((((((Membranous Glomerulonephritides) OR (Membranous Glomerulonephritis)) OR (Membranous Glomerulopathy)) OR (Membranous Nephropathy)) OR (Extramembranous Glomerulopathy)) OR (Membranous Glomerulonephropathy)) OR (Heymann Nephritis)) OR (Idiopathic Membranous Glomerulonephritis)) OR (Idiopathic Membranous Glomerulonephritides)) OR (Idiopathic Membranous Nephropathy))) |
| **Embase ^[[1]](#footnote-1)^** |
| 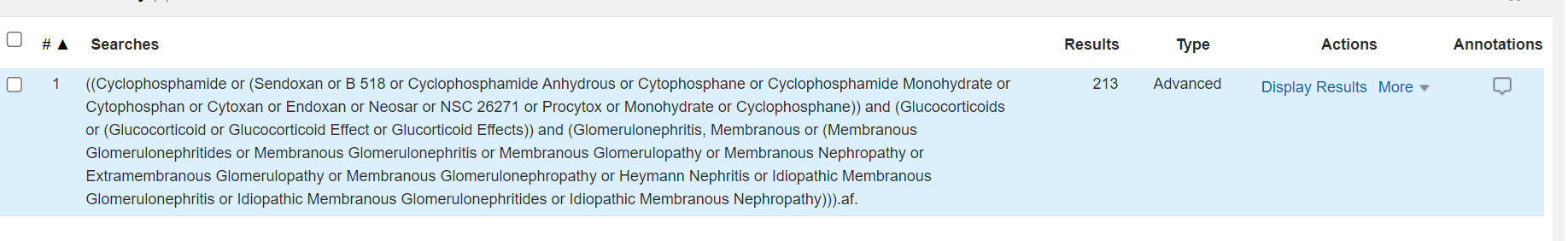 |
| **Cochrane Library^[[2]](#footnote-2)^** |
| **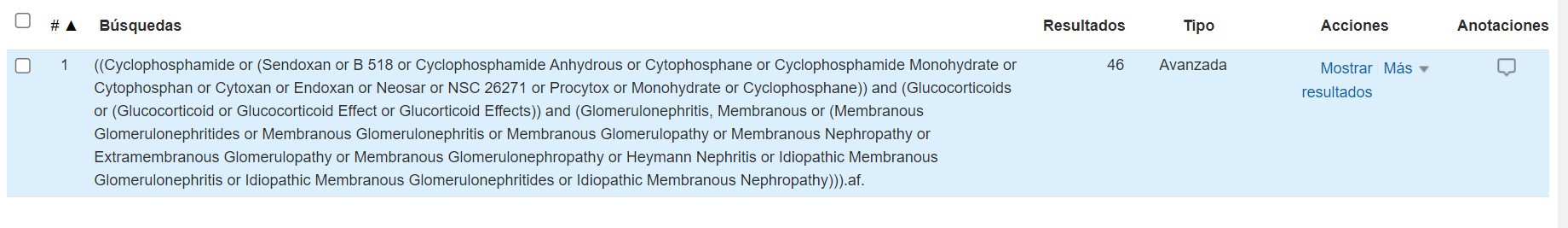** |
| **Web of Science** |
| (((Cyclophosphamide) OR (((((((((((((Sendoxan) OR (B 518)) OR (Cyclophosphamide Anhydrous)) OR (Cytophosphane)) OR (Cyclophosphamide Monohydrate)) OR (Cytophosphan)) OR (Cytoxan)) OR (Endoxan)) OR (Neosar)) OR (NSC 26271)) OR (Procytox)) OR (Monohydrate)) OR (Cyclophosphane))) AND ((Glucocorticoids) OR (((Glucocorticoid) OR (Glucocorticoid Effect)) OR (Glucorticoid Effects)))) AND ((Glomerulonephritis, Membranous) OR ((((((((((Membranous Glomerulonephritides) OR (Membranous Glomerulonephritis)) OR (Membranous Glomerulopathy)) OR (Membranous Nephropathy)) OR (Extramembranous Glomerulopathy)) OR (Membranous Glomerulonephropathy)) OR (Heymann Nephritis)) OR (Idiopathic Membranous Glomerulonephritis)) OR (Idiopathic Membranous Glomerulonephritides)) OR (Idiopathic Membranous Nephropathy))) (Topic) |
| **Wanfang** |
| 全部:(环磷酰胺) and 全部:(糖皮质激素) and 全部:(膜性肾病) |
| **China National Knowledge Infrastructure (CNKI)**  （摘要：环磷酰胺(模糊)）AND（摘要：糖皮质激素(模糊)）AND（摘要：膜性肾病(模糊)） |

1. Via Ovid (https://ovidsp.ovid.com/) [↑](#footnote-ref-1)
2. Via Ovid (https://ovidsp.ovid.com/) [↑](#footnote-ref-2)
