# Supplementary material for: Efficacy and safety of glucocorticoid combined with cyclophosphamide therapy on membranous nephropathy: a systematic review and meta-analysis
Source: Front Pharmacol. 2024 Nov 27;15:1480638. doi: 10.3389/fphar.2024.1480638 (PMC11631627; doi:10.3389/fphar.2024.1480638)
Supplement: Supplementary file 3 [file Table5.DOCX]

Supplementary Material

**Supplementary Table S5** Quality evaluation of the eligible studies with Newcastle–Ottawa scale

| Study | Selection | | | | Comparability | | Outcome | | |
| --- | --- | --- | --- | --- | --- | --- | --- | --- | --- |
|  | Representative-ness | Selection of  non-exposed | Ascertainment  of exposure | Outcome not present at the start | Comparability of most important factors | Comparability on other risk factors | Assessment of outcome | Long enough follow-up (median≥1 year) | Adequacy  (completeness) of follow-up |
| Cui 2017 (1) | * | * | * | * | * | - | * | * | * |
| Liu 2015 (29) | * | * | * | * | * | - | * | - | * |
| Sun 2023 (23) | * | * | * | * | - | - | * | * | * |
| Van den brand 2017(24) | * | * | * | * | - | - | * | * | * |
| Xia 2016 (31) | * | * | * | * | * | - | * | - | * |
| Zou 2019 (25) | * | * | * | * | * | - | * | * | * |

*indicates criterion met; - indicates significant of criterion not met.
