# Supplementary material for: Efficacy and safety of glucocorticoid combined with cyclophosphamide therapy on membranous nephropathy: a systematic review and meta-analysis
Source: Front Pharmacol. 2024 Nov 27;15:1480638. doi: 10.3389/fphar.2024.1480638 (PMC11631627; doi:10.3389/fphar.2024.1480638)
Supplement: Supplementary file 4 [file Table3.DOCX]

Supplementary Material

**Supplementary Table S3** Definitions of complete remission and partial remission in the included studies

| **Study** | **Complete remission** | **Partial remission** |
| --- | --- | --- |
| Austin 2009 (16) | Proteinuria＜0.3 g/day and stable renal function. | Proteinuria＜2.0 g/day proteinuria and＜50% reduction from baseline proteinuria. |
| Chen 2009 (27) | 24h urine protein quantitative < 0.3 g, normal serum albumin, normal serum creatinine. | Provided that renal function is stabilized, serum albumin ≥ 30 g/L, 24 h urine protein quantitatively decreased by ≥ 50% but still did not achieve complete remission. |
| Chen qi 2019 (26) | 24 h urine protein quantification < 0.3 g, normal renal function, Serum albumin > 35 g/L, negative urine protein characterization. | The quantity of 24-hour urinary protein was > 0. 3 g, but < 3. 5 g; or 24-hour urinary protein decreased by 50% compared with the baseline, and renal function was stable (serum creatinine increased < 20% compared with the baseline). |
| Cui 2017 (1) | Proteinuria＜0.3 g/day plus normal creatinine concentration. | Proteinuria 0.3-3.5 g/day and 50% lower than baseline proteinuria, plus stable renal function. |
| Fernández-Juárez 2020 (12) | A reduction of proteinuria from baseline to a value ≤0.3 g/24 h plus stable kidney function (eGFR≥45 ml/min per 1.73 m^2^). | A reduction of proteinuria > 50% from baseline; and a value < 3.5 g/24 h plus stable renal function (eGFR 4≥5 ml/min per 1.73 m^2^). |
| Fu 2012 (28) | 24-hour urinary protein excretion was less than 300 mg, serum albumin was normal, eGFR was stable, and the fluctuation range was less than 15% of the baseline level. | 24 h urinary albumin excretion decreased by more than 50%, above 300 mg, and plasma albumin no less than 30g/L was stable. |
| Guo 2020 (17) | No edema symptom, 24-h urinary protein <0.3 g, and normal serum albumin and serum creatinine levels. | A reduction in edema symptoms and 24-hour urinary protein <3.5 g or a decrease by at least 50% compared with the highest peak value. |
| Hayati 2019 (18) | The protein excretion rate was below 300 mg/d together with  normal renal function on at least three occasions. | The protein excretion level was below 3.5 g/d plus a 50% or greater reduction in protein excretion from previous values together with normal renal function. |
| He 2012 (19) | A decrease in daily urinary protein to 0.3 g or less, plus stable renal function. | A decrease of at least 50% in daily proteinuria (i.e., less than 3.5 g/day of urinary protein) with normal serum creatinine concentration. |
| Liang 2017 (20) | A daily proteinuria level <0.5 g with stable renal function. | Proteinuria of 0.5-3.5 g/day was reduced by no less than 50% of baseline levels with well-preserved renal function. |
| Liu 2015 (29) | The quantity of 24-hour urinary protein was less than 0.3 g, and serum albumin and serum creatinine were normal. | The quantity of 24-hour urinary protein decreased by more than 50% of the basic value, and the serum albumin ≥ 25 g/L, the renal function was stable. |
| Ramachandran 2016 (21) | 24 h urine protein <500 mg/day with normal serum albumin (≥3.5 g/dL) and serum creatinine. | 24 h urine protein ≥500 mg/day, but <2 g/day or <50% of baseline with normal serum albumin (≥3.5 g/dL) and serum creatinine. |
| Ramachandran 2021 (22) | Based on KDIGO guidelines. | Based on KDIGO guidelines. |
| Sun 2023 (23) |  |  |
| Tao 2021 (30) | 24 h urine protein quantification <0.3 g/d, plasma serum protein >35 g/L, normal serum creatinine. | 24 h urine protein quantification <3.5 g/day, plasma serum protein >30 g/L. |
| Van den brand 2017 (24) | A urine protein-to-creatinine ratio＜300 mg/g. | urinary protein-to-creatinine ratio or 24-hour proteinuria from the start of treatment to a level, of 3500 mg/10 mmol creatinine (approximately 3100 mg/g creatinine) or, 3 g/24 h and with a stable serum creatinine. |
| Xia 2016 (31) | Urine protein <0.3 g/day, serum albumin >35 g/L, stable renal function (elevated blood creatinine <15% of basal value). | Urine protein was reduced to 0.3-3.5 g/day or >50% less than before treatment, serum albumin >30 g/L, and renal function stabilized. |
| Xu 2021 (32) | Urinary albumin < 1g/day, serum albumin ≥ 35 g/L, and serum creatinine maintained at normal level. | The amount of urinary protein was ≤ 3.5g/day and decreased by more than 50% compared with the baseline value, and the serum creatinine increased by less than 50% of the baseline value. |
| Xue 2019 (5) | At least two examinations at intervals of more than one week confirmed that the quantity of 24-hour urinary protein was less than 0.3 g/L, the levels of serum creatinine and albumin were normal, and there was no edema. | At least two examinations at an interval of more than one week confirmed that the reduction of 24 h urinary albumin was more than or reached 50% of the baseline value, and less than 3. 5 g/L, the levels of serum creatinine and albumin were close to normal, and there was no edema. |
| Zhang 2016 (33) | The quantity of urinary protein＜0.5 g/L, serum albumin≥35 g/L, the renal function was stable. | Urine protein decreased by >50%, and 0.5-3.5 g/day, serum albumin ≥30 g/L, and renal function was stable. |
| Zhao 2021 (34) | 24-hour urine protein <0.3g, serum albumin >35g/L, normal renal function. | 24-hour urine protein 0.3-3.5g, decrease >50% of the basal value, serum albumin ≥35g/L, stable renal function. |
| Zou 2019 (25) | 24-hour urine protein < 0.3 g, normal serum creatinine concentration. | 24 h urine protein at 0.3–3.5 g and 50% lower than baseline 24 h urine protein with a stable serum creatinine concentration. |
