# Supplementary material for: Efficacy and safety of glucocorticoid combined with cyclophosphamide therapy on membranous nephropathy: a systematic review and meta-analysis
Source: Front Pharmacol. 2024 Nov 27;15:1480638. doi: 10.3389/fphar.2024.1480638 (PMC11631627; doi:10.3389/fphar.2024.1480638)
Supplement: Supplementary file 5 [file Table4.DOCX]

Supplementary Material

**Supplementary Table S4** The characteristics of studies included in this meta-analysis

| **Study** | **Study design** | **Region** | **Male** | | **Age(years)** | | **Follow-up(month)** | **Patients** | | **Population** | **Baseline Proteinuria(g/24h)** | |
| --- | --- | --- | --- | --- | --- | --- | --- | --- | --- | --- | --- | --- |
|  |  |  | **T** | **C** | **T** | **C** |  | **T** | **C** |  | **T** | **C** |
| Austin 2009 (16) | RCT | America | 3 | 1 | 40.14±12.36 | 34.19±13.15 | 12 | 15 | 12 | SMN | 6.39±3.65 | 6.74±3.39 |
| Chen 2009 (27) | RCT | China | 28 | | 55.696±11.390 | | 6 | 9 | 8 | PMN | 3.9±1.6 | 4.0±0.7 |
| Chen qi 2019 (26) | RCT | China | 22 | 6 | 57.625±8.465 | 51.286±15.750 | 6 | 32 | 14 | PMN | 5.018±2.640 | 5.621±2.638 |
| Cui 2017 (1) | Retrospective study | China | 72 | 40 | 48.5±6.2 | 48.4±7.8 | 12 | 117 | 60 | PMN | 6.29±1.10 | 6.54±1.26 |
| Fernández-Juárez 2020(12) | RCT | Spain | 24 | 31 | 56.2±12.0 | 55.2±10.8 | 24 | 43 | 43 | PMN | 7.86±4.99 | 8.64±3.76 |
| Fu 2012 (28) | RCT | China | 8 | 9 | 42.7±14.5 | 43.1±11.9 | 36 | 13 | 13 | PMN |  |  |
| Guo 2020 (17) | RCT | China | 15 | 17 | 49.20±11.59 | 49.13±13.71 | 24 | 30 | 30 | PMN | 6.45±1.58 | 6.82±2.28 |
| Hayati 2019 (18) | RCT | Iran | 9 | 10 | 38.69±6.52 | 38.11±7.27 | 12 | 13 | 17 | PMN | 8.78±8.74 | 5.24±1.66 |
| He 2012 (19) | RCT | China | 19 | 20 | 47.2±13.4 | 45.4±11.5 | 12 | 28 | 28 | PMN | 6.38±2.19 | 6.76±2.33 |
| Liang 2017 (20) | RCT | China | 9 | 16 | 53.9±10.4 | 48.2±13.5 | 12 | 28 | 30 | PMN | 6.9±2.2 | 5.9±2.7 |
| Liu 2015 (29) | Retrospective study | China | 20 | 20 | 33-61 | 22-68 | 6 | 24 | 29 | PMN | 13.69±8.70 | 11.38±5.16 |
| Ramachandran 2016 (21) | RCT | India | 20 | 27 | 40.80±10.64 | 38.66±1.91 | 12 | 35 | 35 | PMN | 5.44/1.35 | 6.76/2.14 |
| Ramachandran 2021 (22) | RCT | India | 20 | 27 | 40.0±10.6 | 38.6±11.3 | 72 | 35 | 35 | PMN | 5.22±2.42 | 6.44±3.86 |
| Sun 2023 (23) | Retrospective study | China | 34 | 71 | 49.70±11.99 | 44.34±16.45 | 12 | 135 | 170 | PMN |  |  |
| Tao 2021 (30) | RCT | China | 27 | 27 | 59.7±12.4 | 59.4±12.8 | 6 | 41 | 43 | SMN | 6.22±2.65 | 5.97±2.74 |
| Van den brand 2017 (24) | Retrospective study | Italy | 78 | 72 | 55.3(12.7) | 51.5(15.9) | 40 | 103 | 100 | PMN |  |  |
| Xia 2016 (31) | Retrospective study | China | 14 | 21 | 44.4±10.1 | 41.2±14.1 | 6 | 23 | 28 | PMN | 7.26±2.06 | 7.54±2.70 |
| Xia wq 2016 (31) | Retrospective study | China | 24 | 21 | 45.2±11.1 | 41.2±14.1 | 6 | 39 | 28 | PMN | 7.50±2.55 | 7.54±2.70 |
| Xu 2021 (32) | RCT | China | 39 | 19 | 58.17±33.40 | 48.29±44.95 | 9 | 62 | 33 | PMN | 7.9±3.7 | 7.9±4.2 |
| Xue 2019 (5) | RCT | China | 44 | | 47.05±6.05 | | 12 | 40 | 40 | PMN | 6.47±1.26 | 6.38±1.30 |
| Zhang 2016 (33) | RCT | China | 22 | 21 | 49.3±5.2 | 48.7±5.5 | 12 | 35 | 35 | PMN |  |  |
| Zhao2021 (34) | RCT | China | 21 | 19 | 59.83±11.26 | 55.72±10.43 | 18 | 35 | 30 | PMN | 8.16±1.47 | 8. 82±1.53 |
| Zou 2019 (25) | Retrospective study | China | 83 | 34 | 46.84±11.67 | 40.46±17.16 | 18 | 142 | 61 | PMN | 8.68/0.41 | 8.89/0.17 |

**Supplementary Table S4-continue**

| **Study** | **Treatment** | |
| --- | --- | --- |
|  | T | C |
| Austin 2009 (16) | GC: Starting dose, oral, initiated at 40 mg/m^2^ body surface area.  CTX: Every other month six doses, ranging from 0.5 to 1.0 g/m^2^ body surface area. | GC: Starting dose, oral, initiated at 40 mg/m^2^ body surface area.  CsA: Initiated at a dose of 200 mg/m² of body surface area [approximately 5 mg/kg of body weight] per day, administered in two equal doses at 12-hour intervals over a period of 11 months. |
| Chen 2009 (27) | GC: Starting dose, oral prednisone 15-60 mg/d.  CTX: 750 mg/m^2^ body surface area for 6 months. | GC: Starting dose, oral prednisone 15-60 mg/d.  TAC: First dose, 0.1 mg/(kg·d), taken 2 times 1 hour before meals, for 6 consecutive months. |
| Chen qi 2019 (26) | GC: Starting dose, oral prednisone dose of 0.5mg/kg/day.  CTX: 0.5-0.75g/m^2^ intravenously once a month for a total dose of 7.5-11.5 g, 6 months. | TAC: Starting dose is 0.05 mg/(kg·d), taken twice daily, 1 hour before meals. Blood drug concentration is tested after 3 days of use. Continued use for 6 months, followed by monthly regular monitoring of blood concentration of tacrolimus, with a target range of 3 to 5 μg/L. |
| Cui 2017  (1) | GC: Starting dose, oral prednisone dose 1.0 mg/kg/day.  CTX: Oral, dose of 100 mg for 3 months. | GC：Starting dose, oral prednisone dose 1.0 mg/kg/day.  TAC: The initial dose is 0.05 mg kg-1 day-1, divided into two doses with a 12-hour interval. |
| Fernández-Juárez 2020 (12) | GC: Methylprednisolone treatment at months 1, 3, and 5, 1g intravenous on days 1, 2, and 3, then 0.5 mg/kg/day orally from day 4 through day 30.  CTX: At months 2, 4, and 6, oral cyclophosphamide treatment (1.0-2.0 mg/kg/day for 30 days). | TAC：Oral administration of tacrolimus (0.05 mg/kg/day) to achieve a target blood drug concentration of 5-7 ng/ml, for a duration of 6 months.  RTX：Intravenous injection of rituximab (1 g), with a reduction of tacrolimus dosage by 25% monthly, with complete discontinuation of the medication at the end of the 9th month. |
| Fu 2012 (28) | GC: Starting dose, oral prednisone dose 1mg/kg/day.  CTX: 1 g intravenous once a month for 6 months. | GC: Starting dose, oral prednisone dose 1mg/kg/day.  MMF: The initial dose is 2 g/day taken orally in two divided doses. After 6 months, the dose is reduced to 1.5 g/day, then reduced to 1 g/day after 18 months, further reduced to 0.5 g/day after 30 months, and gradually discontinued. |
| Guo 2020 (17) | GC: Starting dose, oral prednisone dose 1mg/kg/day  CTX: 0.30-0.40 g (m^2^)^-1^ every 2 weeks. | GC：Starting dose, oral prednisone dose 1mg/kg/day.  LEF：20 mg·d^-1^ |
| Hayati 2019 (18) | GC: Methylprednisolone pulse therapy with 1 g intravenous daily for 3 days without oral prednisone followed by oral prednisone 0.5 mg/kg/d for 27 days.  CTX: 1.5-2mg /kg/d at months 2, 4, 6 | GC: Methylprednisolone pulse therapy with 1 g intravenous daily for 3 days without oral prednisone followed by oral prednisone 0.5 mg/kg/d for 27 days.  MMF: Mycophenolate mofetil 2 g/day, administered in two divided doses, for a duration of 6 months. |
| He 2012 (19) | GC: Starting dose, oral prednisone dose 1mg/kg/day.  CTX: Intravenous (750 mg/m^2^ body surface) every 4 weeks for 24 weeks. | GC: Starting dose, oral prednisone dose 1mg/kg/day.  TAC: The dose of TAC is 1 mg/day, continued for 1 week. Subsequently, TAC is administered alternately at doses of 1 mg/day and 2 mg/day. The 2 mg dose of TAC is divided into two equal doses with a 12-hour interval. 1 mg daily and 2 mg daily are administered alternately. |
| Liang 2017 (20) | GC: Starting dose, oral prednisone dose 1mg/kg/day.  CTX: 0.5-0.75g/m^2^ intravenously once a month for the first 6 months and every 2-3 months in later years, cumulative dose of 150 mg/kg. | TAC: The initial dose is 0.05-0.1 mg/kg/day, divided into two doses with a 12-hour interval, without the use of corticosteroids. Adjust the dose based on the target blood trough concentration of 5-10 ng/ml for the first 6 months, followed by a decrease from 4 ng/ml to 6 ng/ml over the next 3 months. Then gradually reduce the dose and discontinue the medication by the end of 12 months. |
| Liu 2015 (29) | GC: Starting dose, oral prednisone dose 1mg/kg/day.  CTX: 750 mg/m^2^ body surface area static spotting, once a month, up to a cumulative dose of 6-8 g. | GC: Starting dose, oral prednisone dose 1mg/kg/day.  TAC: The initial oral dose of tacrolimus is 0.05 mg/(kg·d), taken twice daily with a 12-hour interval, either 1 hour before a meal or 2 hours after a meal, for a continuous duration of 6 months. |
| Ramachandran 2016 (21) | GC: Intravenous methylprednisolone 1 g/day, followed by oral prednisolone 0.5 mg/kg/day for 27 consecutive days in months 1, 3, and 5.  CTX: Oral CTX 2 mg/kg/day at months 2, 4 and 6. | GC: Oral prednisolone at a dose of 0.5 mg/kg/day, continued for 6 months.  TAC: Oral administration of TAC at a dose of 0.1 mg/kg/day, divided into two doses, continuously for one year. Maintain trough concentrations at 5-10 ng/mL for the first 6 months, and at 4-8 ng/mL for the following 6 months. |
| Ramachandran 2021 (22) | GC: Methylprednisolone 1 g intravenously on days 1-3 and oral prednisolone 0.5 mg/kg on days 4-30, months 2, 4 and 6.  CTX: Oral CTX 2-2.5mg/kg/day at months 2, 4 and 6. | GC: Oral prednisolone (0.5 mg/kg daily, continued for 6 months).  TAC: Receive an adequate amount of TAC to maintain a trough concentration of 5-10 ng/ml (specifically 7.46 ng/ml) for a duration of 12 person-months. |
| Sun 2023 (23) | GC: Starting dose, oral prednisone acetate 1mg/kg/day.  CTX: Intravenous administration of 12-15 mg/kg, approximately 0.6-1.2 g, once a month for at least 6 months. | GC: Starting dose, oral prednisone acetate 1mg/kg/day.  CsA: Oral starting dose is 3-5 mg/(kg·day), blood drug concentration is 762 ng/mL.  TAC: Oral administration of TAC at a dose of 0.05-0.1 mg/kg/day. Tacrolimus plasma concentrations are measured 1-2 weeks later and maintained at 5-10 ng/mL. Treatment should last for at least 6 months. |
| Tao 2021 (30) | GC: Monthly dexamethasone sodium phosphate injection 10 mg intravenous for 1 day each time for 3 days.  CTX: 0.4 g intravenous once daily for 2 consecutive days, 0.8 g per month, stopping when the total reaches 8 g. | GC: Start with prednisone 1 mg·kg^-1^·d^-1^, after 8 weeks of treatment.  TAC: Tacrolimus capsules 0.05 mg·kg^-1^·d^-1^, monitor and maintain the blood concentration of tacrolimus at 3-5 μg/L. |
| Van den brand 2017 (24) | GC: Methylprednisone pulses (1 g) were infused on days 1 - 3, 61-63, and 121-123, with oral prednisone 0.5 mg/kg every other day for 5 months and then tapered.  CTX: Oral cyclophosphamide (1.5 mg/kg day) 6-12 months; Cumulative dose (up to 36 g). | RTX: Treatment with intravenous infusion of 375 mg/m^2^ of RTX four times a week. |
| Xia 2016 (31) | GC: Intravenous methylprednisolone 0.5g/d × 3 d followed by oral prednisone 0.4 mg/kg/day× 27 d at 1, 3, and 5 months of age.  CTX: Oral cyclophosphamide 100mg/day×30day, 2nd, 4th, 6th month. | GC: Start with prednisone initial dose of 0.5mg·kg^-1^·d^-1^, taken orally in the morning, gradually reduce to maintain at 15mg/d after relief.  CsA: Start cyclosporine A with an initial dose of 3mg·kg^-1^·d^-1^, taken orally twice a day, monitor CsA blood concentration, and maintain it between 100-200ug/L |
| Xia wq 2016 (31) | GC: Starting dose, oral prednisone 0.8mg/kg/day, max 60mg/day.  CTX: 0.75 g/m^2^ IV drip per month. | GC: Start with prednisone initial dose of 0.5mg·kg^-1^·d^-1^, taken orally in the morning, gradually reduce to maintain at 15mg/d after relief.  CsA: Start cyclosporine A with an initial dose of 3mg·kg^-1^·d^-1^, taken orally twice a day, monitor CsA blood concentration, and maintain it between 100-200ug/L |
| Xu 2021 (32) | GC: Starting dose, oral prednisone 0.5-1.0mg/kg/d.  CTX: 0.50-0.75 g/m^2^ intravenous shock therapy, 6 months. | GC: Oral prednisone or prednisolone, initial dose is 0.5 mg/(kg·d).  CsA: CsA 3-5 mg/(kg·d), orally in two divided doses, maintain CsA trough concentration at 100-200 ng/mL, and peak concentration at 800-1000 ng/mL. |
| Xue 2019 (5) | GC: Starting dose, oral prednisone 0.5-1.0 mg/kg/day.  CTX: 500-1000 mg/m^2^ intravenously, once a week for 2 weeks, repeat after half a month intervals for 3-4 times. | CsA: The dosage of cyclosporine is 3-5 mg/(kg·d), taken orally in two divided doses. |
| Zhang 2016 (33) | GC: Starting dose, oral prednisone 0.5 mg/kg/day.  CTX: 0.8-1.0g intravenous drip, once a month, continued treatment for 6 months and then given every 3 months 0.8g each time, a total of 3 times, until the total amount reaches 7.2-8.4g and then discontinued. | GC: Starting dose, oral prednisone 0.5 mg/kg/day.  CsA: The dosage of cyclosporine is 3-5 mg/(kg·d), taken orally in two divided doses. |
| Zhao2021 (34) | GC: Starting dose, oral prednisone 0.5 mg/kg/day.  CTX: Intravenous injections of 750 mg/m^2^ body surface area each time, every 4 weeks, with a cumulative dose not to exceed 8 g, for a total of 12 months of treatment. | GC: Starting dose, oral prednisone 0.5 mg/kg/day.  TAC: The initial dose of tacrolimus is 0.05 mg/(kg·d), taken in two divided doses (12-hour intervals, on an empty stomach), for continuous treatment for 6 months. |
| Zou 2019 (25) | GC: Starting dose, oral prednisone 0.8-1.0 mg/kg/day, cumulative dose not greater than 8g;  CTX: 750mg/m^2^ intravenous every 4 weeks for 6-12 months, cumulative dose not greater than 8g; | GC: Oral administration of corticosteroids at 0.5 mg/kg/day (not less than 30 mg/day) for 8 weeks, followed by a gradual tapering (decreasing by 5 mg every 4 weeks) to a maintenance dose of 10 mg/day.  TAC: The dosage of TAC is 0.05 mg/kg/day (not exceeding 0.15 mg/kg/day), divided into two equal doses with a 12-hour interval. |

RCT, randomize controlled trials；T, treatment group; C, control group; RTX, rituximab; PMN, Primary membranous nephropathy; SMN, Secondary membranous nephropathy; CR, Complete remission; TR, Total remission; GC, Glucocorticosteroid; CsA, Cyclosporine; TAC, Tacrolimus; MMF, Mycophenolate mofetil; LEF, Leflunomide; CTX, Cyclophosphamide.
